# Paeonol attenuates inflammation by targeting HMGB1 through upregulating miR-339-5p

**DOI:** 10.1038/s41598-019-55980-4

**Published:** 2019-12-18

**Authors:** Liyan Mei, Meihong He, Chaoying Zhang, Jifei Miao, Quan Wen, Xia Liu, Qin Xu, Sen Ye, Peng Ye, Huina Huang, Junli Lin, Xiaojing Zhou, Kai Zhao, Dongfeng Chen, Jianhong Zhou, Chun Li, Hui Li

**Affiliations:** 1 School of Basic Medical Sciences, Guangzhou University of Chinese Medicine, Guangzhou, Guangdong Province 510006 China; 2 Guangdong-Hongkong-Macau Institute of CNS Regeneration, Jinan University, Guangzhou, 510632 China; 3 School of Basic Medical Sciences, Guiyang University of Chinese Medicine, Guiyang, Guizhou Province 550025 China; 4 School of Nursing Sciences, Guangzhou University of Chinese Medicine, Guangzhou, Guangdong Province 510006 China

**Keywords:** Inflammation, Sepsis

## Abstract

Sepsis is a life-threatening disease caused by infection. Inflammation is a key pathogenic process in sepsis. Paeonol, an active ingredient in moutan cortex (a Chinese herb), has many pharmacological activities, such as anti-inflammatory and antitumour actions. Previous studies have indicated that paeonol inhibits the expression of HMGB1 and the transcriptional activity of NF-κB. However, its underlying mechanism is still unknown. In this study, microarray assay and reverse transcription-quantitative polymerase chain reaction **(**RT-qPCR) results confirmed that paeonol could significantly up-regulate the expression of miR-339-5p in RAW264.7 cells stimulated by LPS. Dual-luciferase assays indicated that miR-339-5p interacted with the 3′ untranslated region (3′-UTR) of HMGB1. Western blot, immunofluorescence and enzyme-linked immunosorbent assay (ELISA) analyses indicated that miR-339-5p mimic and siHMGB1 both negatively regulated the expression and secretion of inflammatory cytokines (e.g., HMGB1, IL-1β and TNF-α) in LPS-induced RAW264.7 cells. Studies have confirmed that IKK-β is targeted by miR-339-5p, and we further found that paeonol could inhibit IKK-β expression. Positive mutual feedback between HMGB1 and IKK-β was observed when we silenced HMGB1 or IKK-β. These results indicated that paeonol could attenuate the inflammation mediated by HMGB1 and IKK-β by upregulating miR-339-5p expression. In addition, we constructed CLP model mice by cecal ligation and puncture. Paeonol was used to intervene to investigate its anti-inflammatory effect *in vivo*. The results showed that paeonol could improve the survival rate of sepsis mice and protect the kidney of sepsis mice.

## Introduction

Sepsis is a systemic inflammatory response syndrome (SIRS) caused by infection and associated with high rates of morbidity and mortality^1^. It has been reported that sepsis is mainly caused by inflammation and eventually leads to organ dysfunction^2^. Inflammation can occur in any tissue affected by infection, burning, toxins, etc^3^. A proper inflammatory process is essential for injury healing, while the immunosuppressive reaction is also indispensable for keeping inflammation from becoming too severe^4^. However, once the balance between the anti**-/**proinflammatory reactions is destabilized, proinflammatory cytokines may be overexpressed by leukocytes or lymphocytes and subsequently lead to healthy tissue damage, a process termed the inflammatory cascade. The inflammatory cascade is closely related to numerous human diseases, such as Parkinson’s disease, organ failure, cancer, and especially sepsis^5–7^.

High mobility group box 1 (HMGB1), a highly conserved non-histone DNA-binding protein located in the nucleus^8^, is secreted into the cytoplasm when a tissue is damaged due to infection^9^. As a late proinflammatory mediator, extracellular HMGB1 mediates the inflammatory cascade^10^ and promotes the production of inflammatory cytokines (e.g., TNF-α and IL-1β) by binding to multiple receptors such as RAGE and TLRs^11–13^. Studies have shown that HMGB1-specific antibodies and HMGB1 antagonists can significantly reduce the inflammatory responses and mortality associated with sepsis. Numerous HMGB1-targeted therapies against inflammation have been proposed, but the most promising therapies are still in preclinical or clinical development^14–19^. Given the importance of HMGB1 in inflammatory processes, potential HMGB1-targeted therapies and medicines may be essential for clinical therapeutic strategies.

MicroRNAs (miRNAs) are small non-coding RNAs with a length of 21–23 nucleotides. MiRNAs regulate gene expression by suppressing translation or inducing mRNA cleavage by binding to the 3′ untranslated region (3′-UTR) site of target mRNAs^20^. To date, the close associations between miRNAs and inflammation have been shown by a large number of studies^21–25^. MiRNAs (e.g., miR-142-3p, miR-181a, miR-126, and miR-155) have also been reported to regulate inflammation by targeting HMGB1^23,24,26–28^.

Traditional Chinese medicine has a long history of treating infectious diseases and has a marked curative effect. Paeonol (2′-hydroxy-4′-methoxyacetopheone) is one of the active components in the traditional Chinese medicine moutan cortex^29^. Studies have confirmed that paeonol has a wide range of pharmacological activities, including playing an important role in the inhibition of inflammation and producing antitumour and anti-diabetic effects^30–32^. A previous study found that paeonol can downregulate the expression and secretion of the inflammatory factor HMGB1 to treat acute lung injury in an endotoxic shock rat model^33^. However, the mechanism by which paeonol inhibits HMGB1 remains unclear. Given the important role of miRNAs in inflammation, we suspect that miRNAs play a key role in HMGB1 inhibition by paeonol.

Studies have shown that more than 40% of sepsis patients eventually develop into septic acute kidney injury (SIAKI). Acute kidney injury (AKI) is a clinical syndrome caused by various causes of rapid decline in renal function. Clinically, when sepsis complicated with acute kidney injury, the mortality rate of clinical patients can be as high as 70%^34^. A large number of studies have shown that AKI-induced systemic inflammatory reactions cause multiple organ damage, which is a key factor leading to increased mortality in clinical AKI patients. inflammatory factors such as HMGB1 play an important role in systemic inflammatory response^35^. In the pathophysiological process of sepsis, on the one hand, endotoxin directly damages the body, on the other hand, endotoxin induces a series of serious cascade inflammatory reactions, and produces a large number of early and late inflammatory factors such as TNF-α, IL-1β, etc. Inflammatory factors aggravate the cascade inflammatory reactions until induced septic shock, single organ or even multiple organ failure syndrome (MODS)^36^. The complex inflammatory and immune responses induced by endotoxin may be the main factors and mechanisms of acute renal injury in sepsis.

In the present study, miRNA array analyses including wild-type RAW264.7 cells, lipopolysaccharide (LPS)-stimulated RAW264.7 cells and paeonol-treated, LPS-stimulated RAW264.7 cells were performed. Differentially expressed miRNAs (e.g., miR-182-5p, miR-339-5p, miR-30a-3p, miR-155-3p, and miR-27a-3p) were identified. Among these miRNAs, miR-339-5p is a cancer-related molecule that participates in multiple cell processes such as proliferation, migration and invasion^37,38^. Additionally, studies have indicated that miR-339-5p can inhibit inflammation by regulating the nuclear transcription factor κB (NF-κB) pathway via targeting IKK-β^39,40^. Since HMGB1 is a significant activator of the NF-κB pathway that functions through binding to an NF-κB dimer (P65 and P50)^10^, this study sought to examine whether miR-339-5p inactivates the NF-κB pathway by downregulating HMGB1 expression and to explore the relationship between miR-339-5p and HMGB1. Further explore the anti-inflammatory effect of paeonol *in vivo*.

Thus, we established an inflammatory cell model with LPS to further study the role of miR-339-5p in HMGB1-mediated inflammation and the way paeonol attenuates inflammation. At the same time, CLP mice model was established to study the anti-inflammatory effect of paeonol *in vivo*. This study may provide potential targets and drug candidates for improving the treatment of inflammatory diseases such as sepsis.

## Results

### Paeonol increased miR-339-5p expression in LPS-stimulated RAW264.7 cells

**Figure 1:**
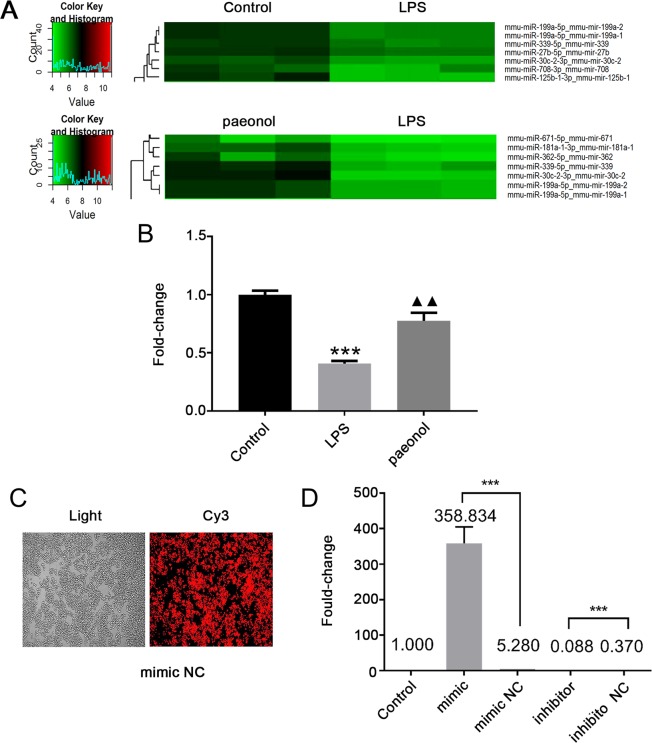
Paeonol increased the miR-339-5p level in LPS-induced RAW264.7 cells. (**A**) MicroRNA levels in the cells in the control group, model group and paeonol group were measured in a microarray assay; partial results with significant changes are shown above. (**B**) Estimations of the miR-339-5p level in the control group, model group and paeonol group were made by RT-qPCR. Note: ***P < 0.001 in comparison with the control group; ^▲▲^*P* < 0.01 in comparison with the model group (LPS). (**C**) The microRNA mimic NC labelled with Cy3 was successfully transfected into RAW264.7 cells. (**D**) MiR-339-5p expression was significantly changed in the mimic and inhibitor groups compared with the control/NC groups after miR-339-5p transfection (RT-qPCR). Note: ***P < 0.001. MiR-339-5p, microRNA-339-5p; NC, negative control.

A microarray assay was used to detect differentially expressed miRNAs among the control group, LPS (model) group and paeonol group. The results, in part, were analysed and illustrated with a cluster diagram. The results showed that the expression of miR-339-5p was decreased in the model group compared with the control group, and the paeonol group demonstrated higher miR-339-5p levels than the model group (*P* < 0.05; Fig. 1A). Reverse transcription-quantitative polymerase chain reaction RT-qPCR showed the same results (*P* < 0.05; Fig. 1B). Considering the relationship between miR-339-5p and inflammation, we selected this miRNA for further research. Immunofluorescence and RT-qPCR analyses (*P* < 0.05; Fig. 1C,D) showed that miR-339-5p was successfully transfected into RAW264.7 cells.

### MiR-339-5p could target the HMGB1 protein and downregulate HMGB1 expression

**Figure 2:**
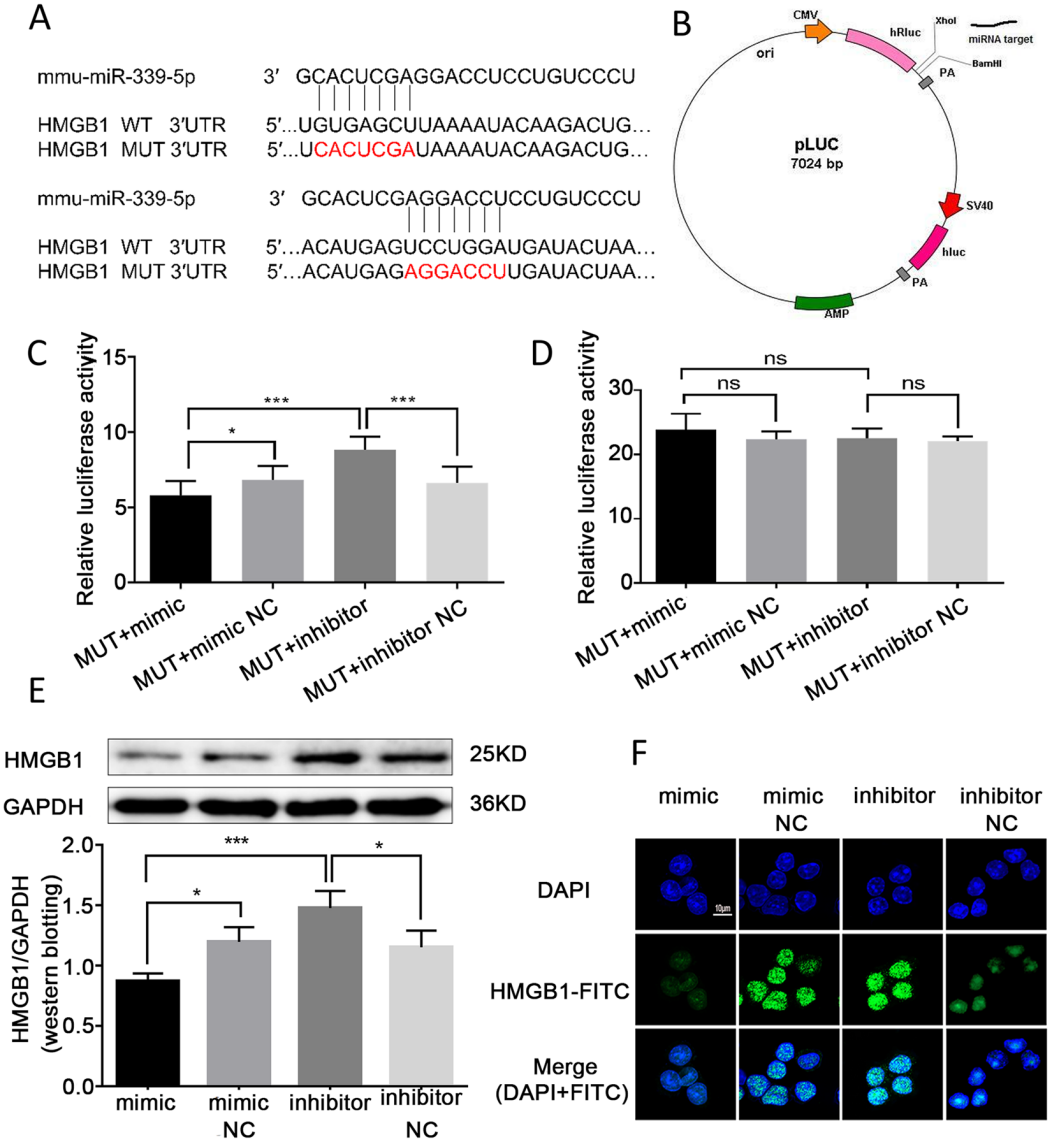
HMGB1 was a direct target of miR-339-5p. (**A**) The target sites of miR-339-5p in the HMGB1 sequence were predicted by bioinformatic software. (**B**) The construction profile of the pLUC-HMGB1 vector, which contained the miR-339-5p target sites in the HMGB1 3′-UTR, is presented in the diagram. (**C,D**) The relative luciferase activity in RAW264.7 cells was measured after the co-transfection of the miR-339-5p mimic, mimic NC, inhibitor or inhibitor NC with HMGB1-WT-3′-UTR or HMGB1-MUT-3′-UTR. (**E**) The expression of the target protein HMGB1 in RAW264.7 cells was analysed by Western blotting. (**F**) The expression of the target protein HMGB1 in RAW264.7 cells was analysed by immunofluorescence staining (magnification, 400×). Note: *P < 0.05, ***P < 0.001.

To determine whether miR-339-5p targets the HMGB1 protein, we predicted the target site of miR-339-5p in the HMGB1 sequence with bioinformatics software (Fig. 2A). HMGB1 luciferase reporter vectors, which contained a pLUC-WT-HMGB1 or pLUC-MUT-HMGB1 sequence vector of the miR-339-5p binding site, were constructed (Fig. 2B). A miR-339-5p mimic, miR-339-5p inhibitor or the appropriate negative control (NC) was co-transfected with one of the luciferase reporters. The results showed that compared with co-transfection with the negative controls, co-transfection with pLUC-WT-HMGB1 and the miR-339-5p mimic inhibited the relative luciferase activity of the HMGB1 3′-UTR, while co-transfection with pLUC-WT-HMGB1 and the miR-339-5p inhibitor promoted luciferase activity (*P* < 0.05; Fig. 2C). In addition, the pLUC-MUT-HMGB1 vector and miR-339-5p mimic, miR-339-5p inhibitor or appropriate negative control were co-transfected. The results indicated that compared with co-transfection including the negative control, co-transfection with pLUC-MUT-HMGB1 and the miR-339-5p mimic did not change the HMGB1 3′-UTR luciferase activity. There was also no significant difference in the luciferase activity of the HMGB1 3′-UTR between co-transfection with pLUC-MUT-HMGB1 and the miR-339-5p inhibitor and co-transfection including the negative controls (*P* < 0.05; Fig. 2D). The results indicated that miR-339-5p was able to bind to the HMGB1 3′-UTR. Further experiments confirmed that miR-339-5p inhibited the expression of the target protein HMGB1. We transfected the miR-339-5p mimic, the inhibitor and their negative controls into RAW264.7 cells. Western blotting and immunofluorescence results showed that the protein expression of HMGB1 was reduced in the mimic group compared with the mimic negative control group. Compared with the inhibitor negative control, the miR-339-5p inhibitor induced HMGB1 expression (*P* < 0.05; Fig. 2E,F). These results confirmed that miR-339-5p specifically targets the HMGB1 protein and downregulates HMGB1 expression.

### MiR-339-5p and paeonol both inhibit the expression of proinflammatory cytokines in LPS-stimulated RAW264.7 cells at 12 h and 24 h time points

**Figure 3:**
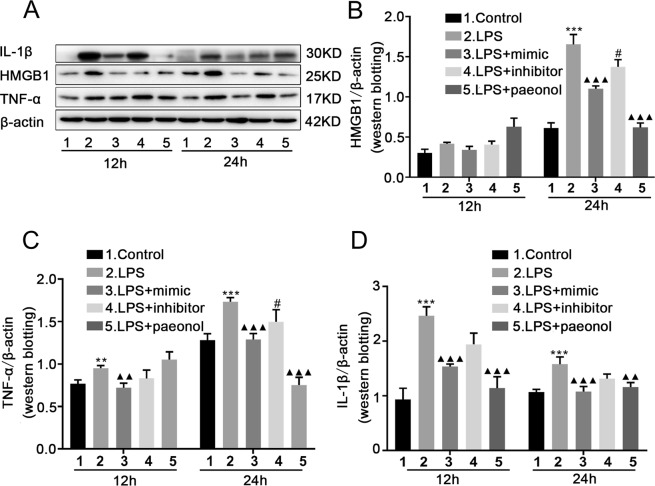
The effects of miR-339-5p and paeonol on LPS-induced RAW264.7 cells at the 12-h and 24-h time points, as measured by Western blotting. (**A–D**) Analysis of the levels of the inflammation-related proteins HMGB1, TNF-α and IL-1β in the (1) normal control, (2) LPS, (3) LPS + miR-339-5p mimic, (4) LPS + miR-339-5p inhibitor and (5) LPS + Paeonol groups of LPS-induced RAW264.7 cells by Western blotting. Note: **P < 0.01 and ***P < 0.001 in comparison with the control group; ^▲▲^*P* < 0.01 and ^▲▲▲^*P* < 0.001 in comparison with the model group (LPS); ^#^*P* < 0.05.

**Figure 4:**
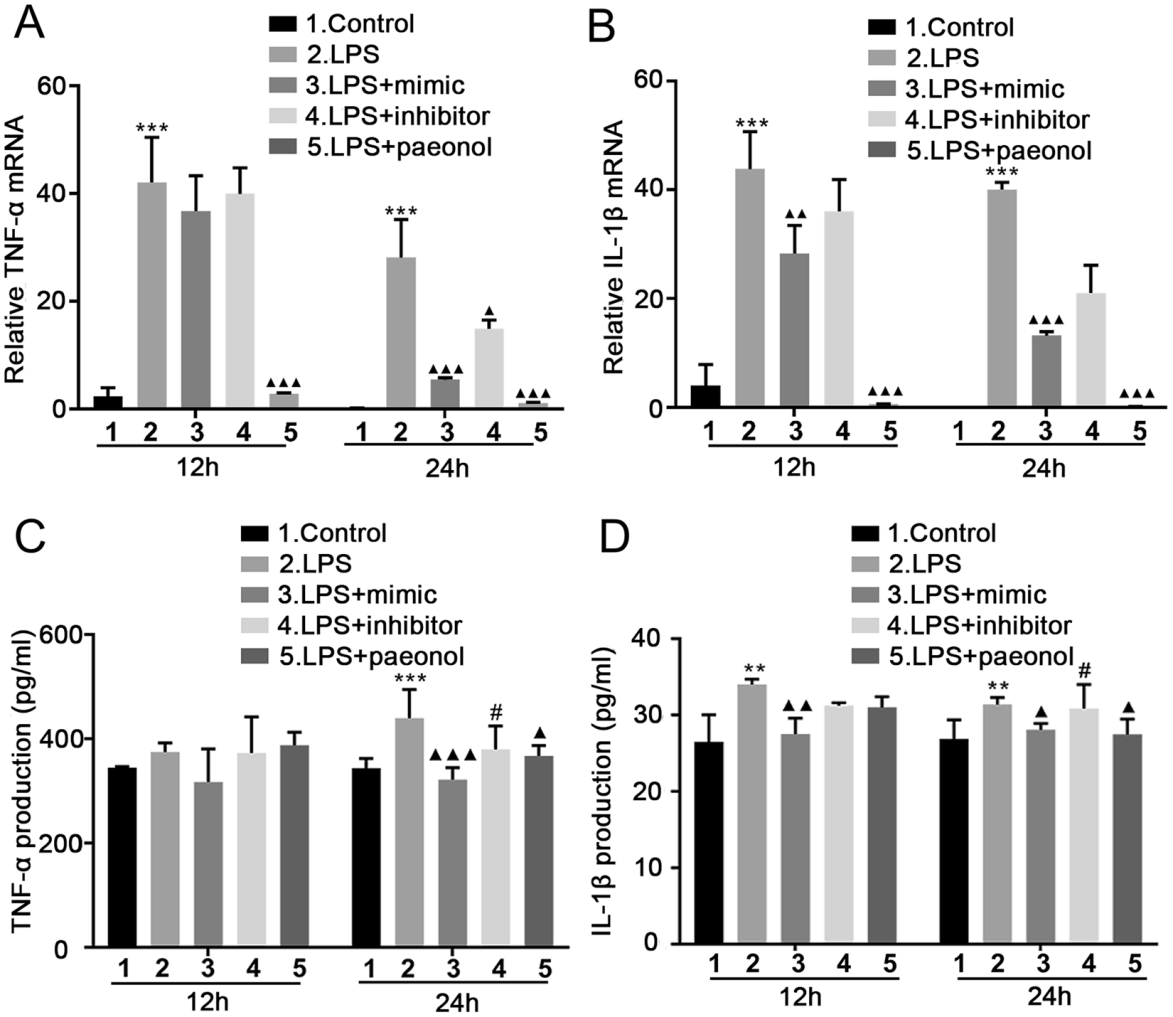
The effects of miR-339-5p and paeonol on LPS-induced RAW264.7 cells at the 12-h and 24-h time points, as measured by RT-qPCR and ELISA. (**A,B**) Analysis of the mRNA production of the inflammation-related cytokines TNF-α and IL-1β by RT-qPCR. (**C,D**) Analysis of the production of the inflammation-related proteins TNF-α and IL-1β by ELISA. Note: **P < 0.01 and ***P < 0.001 in comparison with the control group; ^▲^*P* < 0.05, ^▲▲^*P* < 0.01, and ^▲▲▲^*P* < 0.001 in comparison with the model group (L*P*S); ^#^*P* < 0.05.

To further observe the effects of miR-339-5p and paeonol on inflammatory proteins at 12 h and 24 h time points, we increased miR-339-5p expression, decreased miR-339-5p expression, or applied treatment with paeonol in LPS-stimulated RAW264.7 cells. Western blotting, RT-qPCR and ELISA were utilized to measure the expression of proinflammatory cytokines in RAW264.7 cells. The Western blotting results showed that LPS could significantly upregulate the expression of the proinflammatory cytokines HMGB1, TNF-α and IL-1β, while the miR-339-5p inhibitor had no effect on this phenomenon. However, this phenomenon could be reversed by either paeonol or the miR-339-5p mimic at the 12 h and 24 h time points, and this reversal was significant at 24 h (*P* < 0.05; Fig. 3A–D). The RT-qPCR results also showed that compared with the LPS group, the miR-339-5p and paeonol group exhibited inhibited mRNA production of the inflammatory cytokines TNF-α and IL-1β. Compared to the miR-339-5p inhibitor, the miR-339-5p mimic decreased TNF-α and IL-1β production. However, the effect was more obvious at 24 h (*P* < *0.001*; Fig. 4A,B). In addition, the ELISA results also demonstrated that compared with the LPS group, the miR-339-5p and paeonol groups exhibited inhibited secretion of the inflammatory cytokines TNF-α and IL-1β. Compared to treatment with the miR-339-5p inhibitor, treatment with the miR-339-5p mimic decreased TNF-α and IL-1β production. However, the effect was more obvious at 24 h (*P* < 0.05; Fig. 4C,D). These results demonstrated that miR-339-5p and paeonol exerted anti-inflammatory effects at the 12 h and 24 h time points in LPS-induced RAW264.7 cells, but the anti-inflammatory function at the 24 h time point was more significant. Therefore, we chose 24 h for further experimentation.

### MiR-339-5p inhibited the inflammatory response in an HMGB1-dependent manner

**Figure 5:**
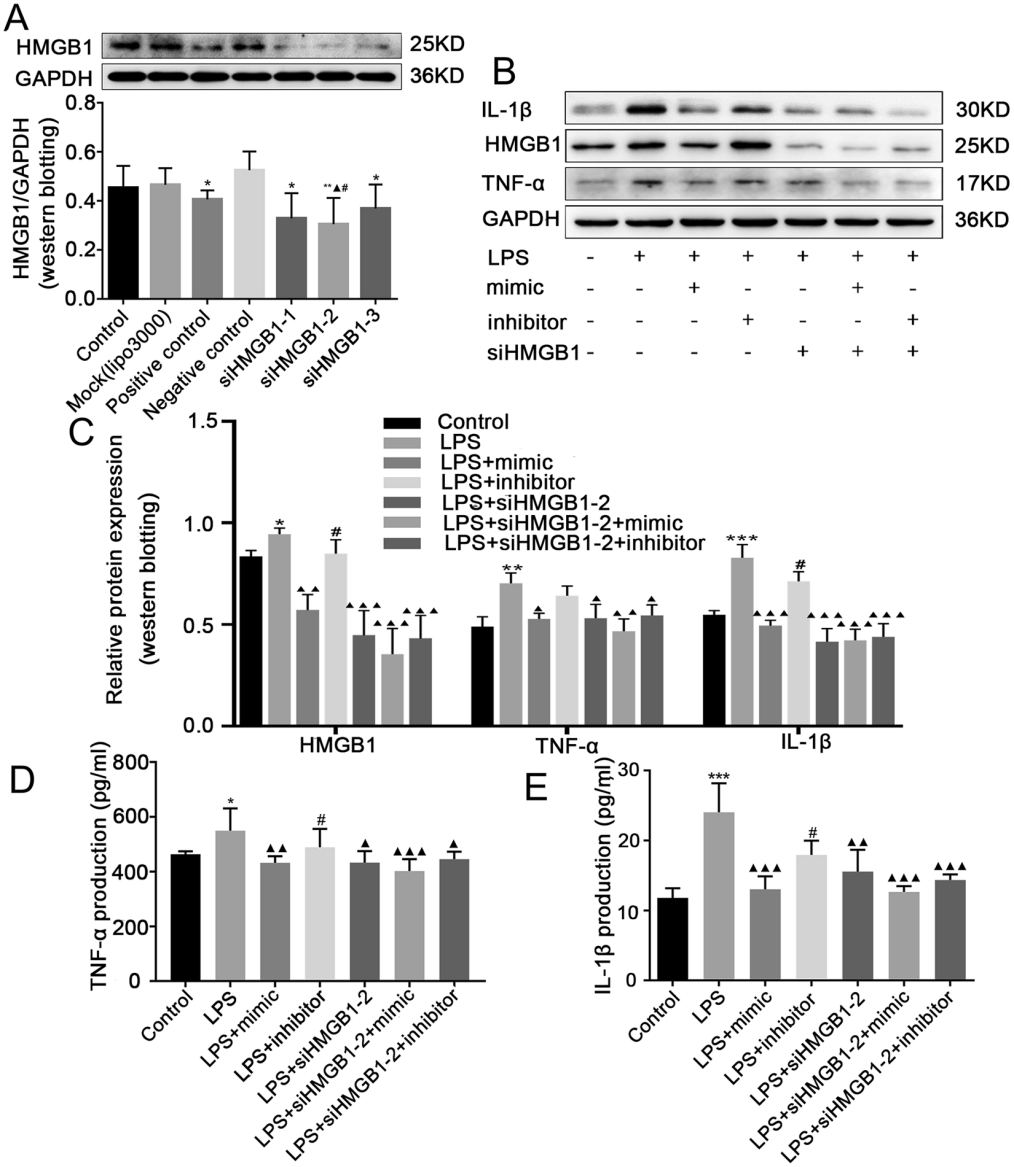
The effects of miR-339-5p and HMGB1-siRNA on LPS-induced RAW264.7 cells at the 24-h time point. (**A**) Detection of the silencing effect of HMGB1-siRNAs (siRNA-1, siRNA-2 and siRNA-3). Note: *P < 0.05 in comparison with the negative control group; ^▲^*P* < 0.05 in comparison with the control group; ^#^*P* < 0.05 in comparison with the Mock group. (**B**,**C**) Analysis of the inflammation-related proteins HMGB1, TNF-α and IL-1β, in the (1) normal control, (2) LPS, (3) LPS + miR-339-5p mimic, (4) LPS + miR-339-5p inhibitor, (5) LPS + siHMGB1-2, (6) LPS + siHMGB1-2 + miR-339-5p mimic and (7) LPS + siHMGB1-2 + miR-339-5p inhibitor groups of LPS-induced RAW264.7 cells by Western blotting. (**D**,**E**) Analysis of the production of the inflammation-related proteins TNF-α and IL-1β by ELISA. Note: **P < 0.01 and ***P < 0.001 in comparison with the control group; ^▲^*P* < 0.05, ^▲▲^*P* < 0.01, and ^▲▲▲^*P* < 0.001 in comparison with the model group (LPS); ^#^*P* < 0.05.

To further investigate whether the anti-inflammatory effect of miR-339-5p involves targeting HMGB1, siHMGB1-1, siHMGB1-2 or siHMGB1-3 was transfected into RAW264.7 cells to determine which siRNA had the best silencing effect. Western blotting results demonstrated that compared with the negative control group, control group and mock group, the siHMGB1-2 group exhibited the best silencing effect (*P* < 0.05; Fig. 5A). Therefore, siHMGB1-2 was selected for subsequent experiments. To study whether the anti-inflammatory effect of miR-339-5p is dependent on HMGB1, siHMGB1-2 and miR-339-5p were co-transfected into LPS-induced RAW264.7 cells. Western blotting results showed that the protein expression of HMGB1, TNF-α and IL-1β was increased in the LPS group. Compared with miR-339-5p inhibitor transfection, miR-339-5p mimic transfection reduced the expression of HMGB1, TNF-α and IL-1β. Compared with the LPS group, the miR-339-5p mimic transfection, siHMGB1-2 transfection, miR-339-5p mimic and siHMGB1-2 co-transfection, and miR-339-5p inhibitor and siHMGB1-2 co-transfection groups exhibited inhibited protein expression of HMGB1, TNF-α and IL-1β. However, there were no significant differences among the siHMGB1-2 transfection, miR-339-5p mimic and siHMGB1-2 co-transfection, and miR-339-5p inhibitor and siHMGB1-2 co-transfection groups (*P* < 0.05; Fig. 5B,C). ELISA analysis showed similar results (*P* < 0.05; Fig. 5D,E).

### The combination of paeonol and miR-339-5p inhibited inflammation more significantly than other treatments

**Figure 6:**
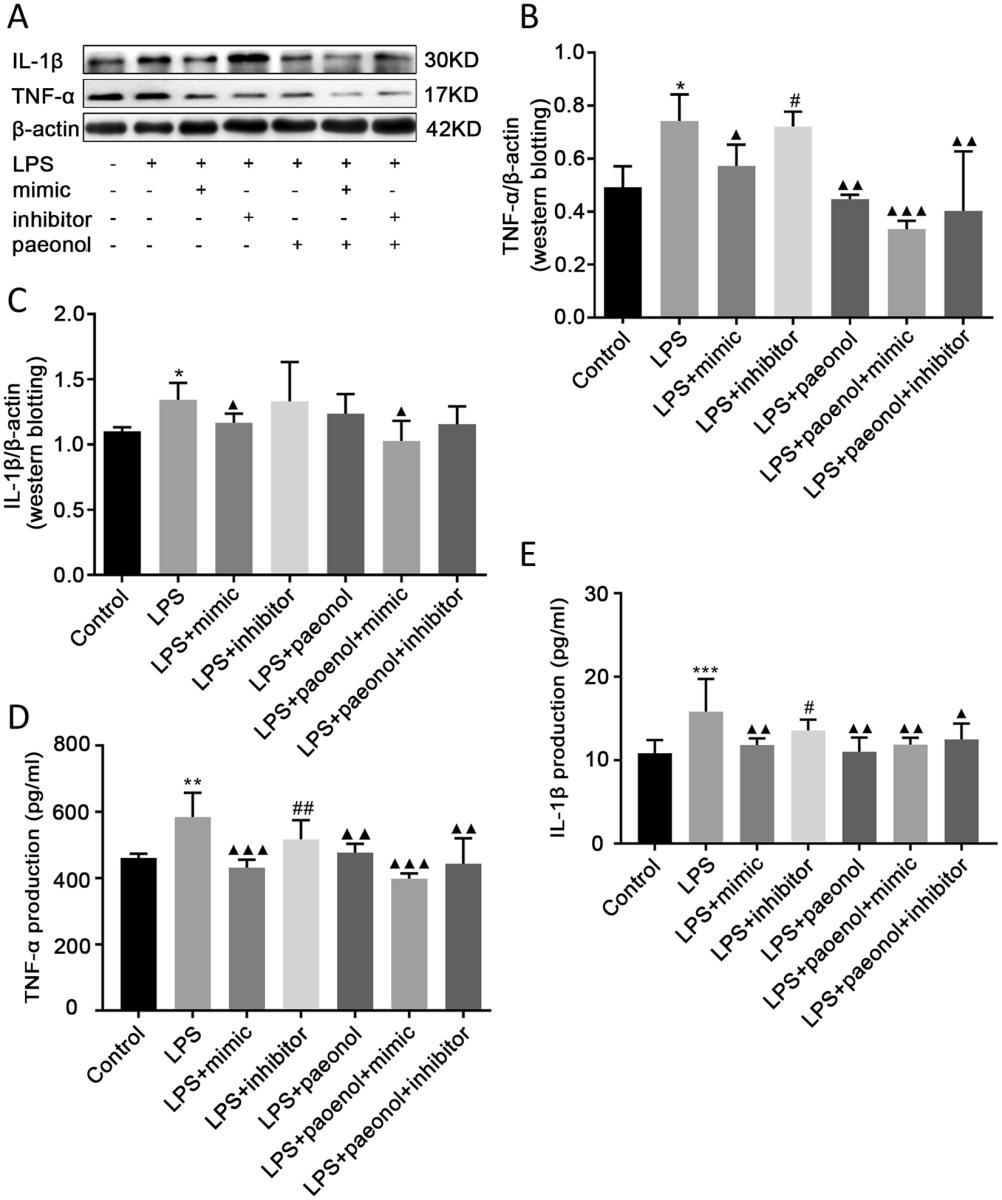
The effects of miR-339-5p and paeonol on LPS-induced RAW264.7 cells at the 24-h time point. (**A–C**) Analysis of the inflammation-related proteins TNF-α and IL-1β in (1) normal control, (2) LPS, (3) LPS + miR-339-5p mimic, (4) LPS + miR-339-5p inhibitor, (5) LPS + Paeonol, (6) LPS + miR-339-5p mimic + Paeonol and (7) LPS + miR-339-5p inhibitor + Paeonol groups of LPS-induced RAW264.7 cells by Western blotting. (**D,E**) Analysis of the production of the inflammation-related proteins TNF-α and IL-1β by ELISA. Note: *P < 0.05, **P < 0.01, and ***P < 0.001 in comparison with the control group; ^▲^*P* < 0.05, ^▲▲^*P* < 0.01^,^ and ^▲▲▲^*P* < 0.001 in comparison with the model group (LPS); ^#^*P* < 0.05 and ^##^*P* < 0.01 in comparison with the miR-339^-^5p mimic group.

To further observe the combined anti-inflammatory effects of paeonol and miR-339-5p, miR-339-5p and paeonol were used to co-treat LPS-induced RAW264.7 cells. Western blot and ELISA analyses showed that the levels of the inflammatory cytokines TNF-α and IL-1β were increased in the LPS group. Compared with the miR-339-5p inhibitor group, the miR-339-5p mimic group exhibited reduced expression of TNF-α and IL-1β. Compared with the LPS group, the miR-339-5p mimic, paeonol, miR-339-5p mimic and paeonol, and miR-339-5p inhibitor and paeonol co-transfection groups exhibited downregulated production of TNF-α and IL-1β, but the combination of paeonol and the miR-339-5p mimic showed the best anti-inflammatory effect (*P* < 0.05; Fig. 6A–E).

### Paeonol inhibited IKK-β, which has a positive effect on HMGB1

**Figure 7:**
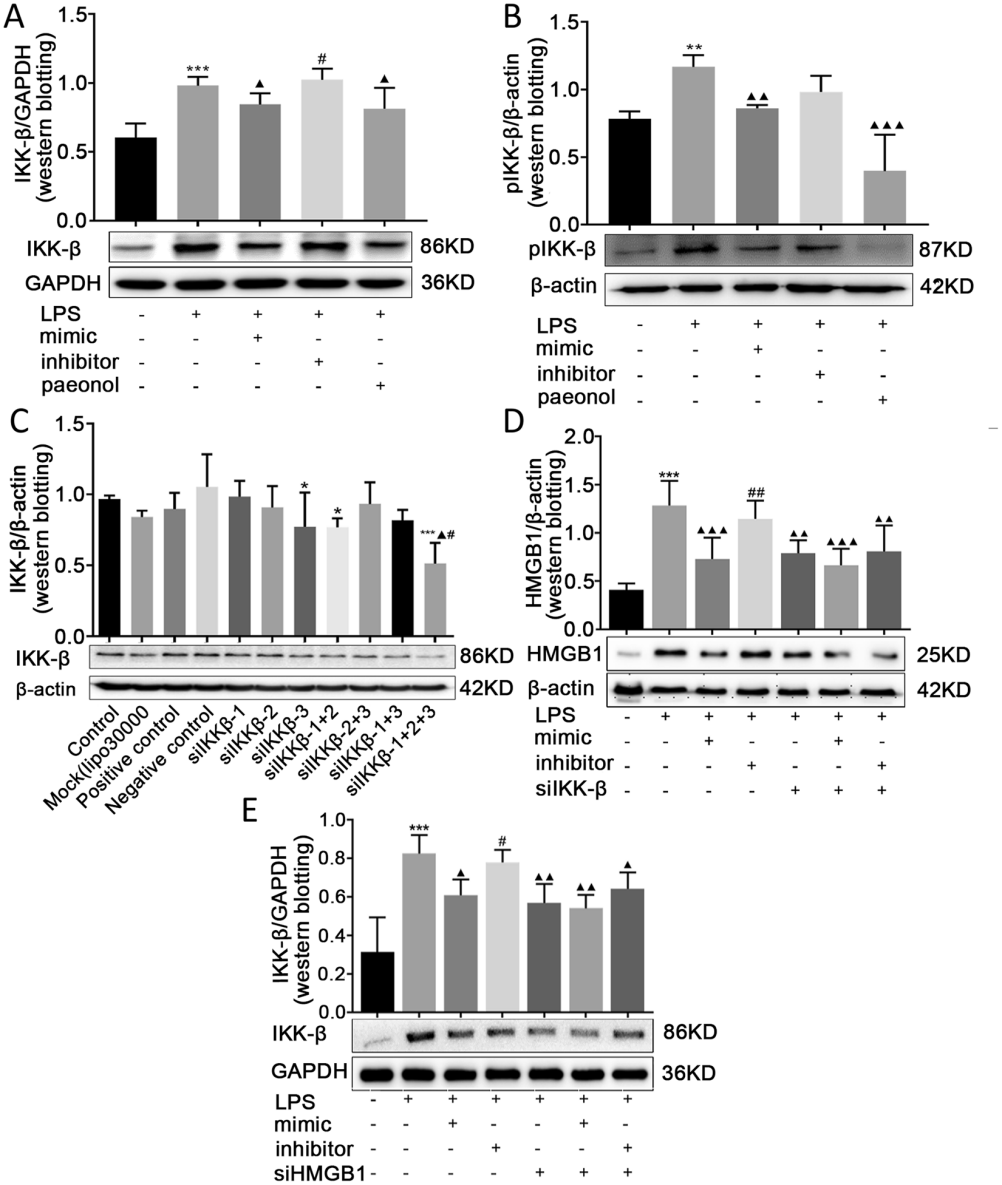
Effects of paeonol on IKK-β in LPS-induced RAW264.7 cells and the relationship between HMGB1 and IKK-β. (**A,B**) The effects of paeonol on IKK-β and pIKK-β expression in LPS-treated cells, assessed by Western blotting. Note:***P < 0.01, ***P < 0.001 in comparison with the control group; ^▲^*P* < 0.05, ^▲▲^*P* < 0.01, and ^▲▲▲^*P* < 0.001 in comparison with the model group (L*P*S); ^#^*P* < 0.05 in comparison with the miR-339-5p mimic group. (**C**) Detection of the silencing effect in the IKK-β-siRNA group compared with that in the negative control group, control group and mock group (Lipofectamine 3000 only). Note: *P < 0.05 and ***P < 0.001 in comparison with the negative control group; ^▲^*P* < 0.05 in comparison with the control group; ^#^*P* < 0.05 in comparison with the mock group. (**D**,**E**) The relationship between HMGB1 and IKK-β in LPS-induced RAW264.7 cells, assessed by Western blotting. Note: ***P < 0.001 in comparison with the control group; ^▲^*P* < 0.05, ^▲▲^*P* < 0.01, and ^▲▲▲^*P* < 0.001 in comparison with the model group (LPS); ^#^*P* < 0.05 and ^##^*P* < 0.01 in comparison with the miR-339-5p mimic group.

Studies have confirmed that miR-339-5p targets IKK-β to inhibit inflammation. Studies have also shown that paeonol increases miRNA-339-5p expression in LPS-stimulated RAW264.7 cells. Thus, Western blotting was performed to determine whether paeonol could downregulate the expression of IKK-β. The Western blotting results showed that paeonol was able to decrease the expression of IKK-β in the LPS-treated cells (*P* < 0.05; Fig. 7A). Compared with the LPS group, the miR-339-5p mimic and paeonol groups exhibited downregulated production of pIKK-β (*P* < 0.001; Fig. 7B), but miR-339-5p mimic and paeonol groups showed down-regulated pIKK-β expression more significantly than down-regulation of IKK-β expression (Fig. 7A,B). The results indicated that paeonol upregulated miR-339-5p expression to inhibit the expression of HMGB1 and IKK-β. We also further studied the relationship between HMGB1 and IKK-β. SiIKK-β-1, siIKK-β-2 and siIKK-β-3 were transfected, individually or in combination, into RAW264.7 cells to assess which siRNA had the best silencing effect. Western blotting results showed that compared with the negative control, control and mock groups, the siIKK-β-1, siIKK-β-2 and siIKK-β-3 combination group showed the best silencing effect (*P* < 0.05; Fig. 7C). SiIKK-β and miR-339-5p were co-transfected into LPS-induced RAW264.7 cells, and the results showed that compared with the LPS group, the siIKK-β group showed downregulated production of HMGB1; however, co-transfection with the miR-339-5p mimic and siIKK-β inhibited HMGB1 more significantly than the transfection of siIKK-β alone (*P* < 0.05; Fig. 7D). The Western blotting results also demonstrated that compared with the LPS group, the siHMGB1 group exhibited decreased expression of IKK-β; however, co-transfection with the miR-339-5p mimic and siHMGB1 inhibited IKK-β more obviously than the transfection of siHMGB1 alone (*P* < 0.05; Fig. 7E).

### Paeonol attenuated CLP-induced septic acute kidney injury

**Figure 8:**
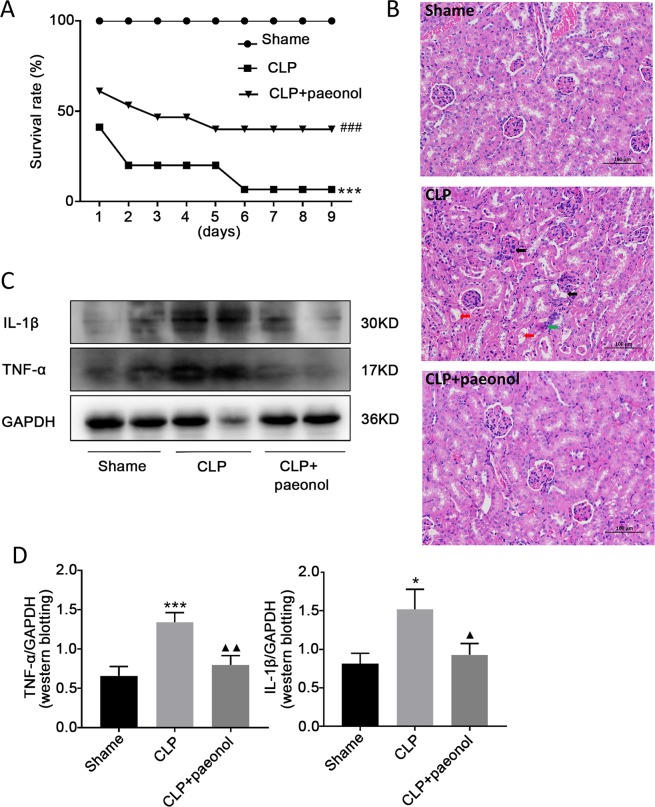
(**A**) The survival rate of C57BL/6 mice after CLP injury with paeonol treatment. Note: ***P < 0.001 in comparison with the shame group; ^###^*P* < 0.001 in comparison with the model group (CLP); (**B**) Morphological changes in the kidney, liver and lung. Tissue sections were stained with hematoxylin and eosin (HE); red arrow in the kidney images indicates cell vacuolization; black arrow indicates that the glomerulus is deformed and blurred with its surroundings; green arrow indicates inflammatory cell infiltration; (**C**,**D**) Western blotting analysis of protein TNF-α and IL-1β expression in kidneys 24 h after CLP with paeonol. Note: *P < 0.05, ***P < 0.001 in comparison with the shame group; ^▲^*P* < 0.05, ^▲▲^*P* < 0.01 in comparison with the model group (CLP).

Using septic mice model of cecal ligation and puncture (CLP), we evaluated the effects of paeonol on acute kidney injury induced by sepsis. Treatment of mice with paeonol after induction of sepsis (0.5 hours) resulted in significant improvement of survival compared with shame group mice (*P* < 0.05; Fig. 8A). Histological evaluation of renal organ specimens in the CLP group showed significant glomerular enlargement deformation, renal inflammation infiltration and vacuolar degeneration histological abnormalities. After the intervention of paeonol, the pathological damage of kidney was obviously improved. Inflammatory cell infiltration, edema, glomerular deformation, necrosis and exfoliation of renal tubular epithelial cells were alleviated. (Fig. 8B). Besides, western blot results showed that compared with the normal control group, the expression of inflammatory factors TNF-α and IL-1β protein in the CLP group was significantly increased. After treatment with paeonol, the protein expression levels of TNF-α and IL-1β were down-regulated to varying degrees relative to the CLP group (*P* < 0.05; Fig. 8C,D).

## Discussion

Previous research suggests that a cytokine-mediated excessive proinflammatory response is a typical feature of sepsis^41^. Therefore, the inhibition of proinflammatory mediators is an important therapeutic strategy for the treatment of various inflammatory diseases, such as sepsis. In this study, an inflammatory cell model was established with LPS-stimulated RAW264.7 cells to investigate the anti-inflammatory properties of paeonol. Our data showed that paeonol could upregulate the expression of miR-339-5p during the inflammatory response and that miR-339-5p could directly target HMGB1 and IKK-β to inhibit inflammation. Further studies confirmed that paeonol inhibited the inflammatory response by upregulating the expression of miR-339-5p, which can negatively regulate HMGB1 and IKK-β. We also found that there was mutual positive feedback between HMGB1 and IKK-β in LPS-induced RAW264.7 cells (Fig. 8). These results represent an in-depth study of the molecular mechanism of the traditional Chinese medicine paeonol in the treatment of inflammation and offer strong proof for the clinical value of this natural medicine. Paeonol and miRNAs may also provide a potential therapeutic strategy for the clinical treatment of inflammatory disease.

It has been confirmed that paeonol inhibits the production of proinflammatory cytokines (TNF-α and IL-1β) and affects the translocation and secretion of the late inflammatory factor HMGB1^42^. The nuclear protein HMGB1 is a late-stage lethal inflammatory factor that is translocated to the extracellular space as a signal for the spread of inflammation^16,43,44^. Studies have shown that therapeutic neutralizing anti-HMGB1 antibodies or other HMGB1 antagonists significantly improve mortality in caecal ligation and puncture (CLP)-induced sepsis^44–47^. Therefore, HMGB1 inhibition is considered a promising therapeutic strategy for inflammatory disease. Our previous study indicated that paeonol significantly inhibits HMGB1 and downregulates inflammatory factor expression in LPS-stimulated RAW264.7 cells^42^. However, the mechanisms underlying how paeonol regulates HMGB1 in the treatment of inflammation are unknown.

Studies have confirmed that a large number of miRNAs are involved in the regulation of the inflammatory process^23,24,27,48,49^. Research on miR-339-5p has mainly focused on tumours, although work on inflammation has also been reported. Zhang *et al*.^50^ found that miR-339-5p can inhibit the activity of IKK-β by regulating the NF-κB signalling pathway, thereby inhibiting inflammation. Newly discovered evidence indicates that miR-339-5p has abnormally low expression, although to different degrees, in solid tumour tissues and tumour cell lines derived from breast cancer^51^, rectal cancer^37^ and other malignant tumours^38,52,53^, suggesting that miR-339-5p may play important roles in the occurrence and development of the aforementioned malignant tumours. Although miR-339-5p expression has been reported in inflammation, research has mainly focused on the NF-κB signalling pathway, and miR-339-5p has not been studied in the context of its ability to directly target the transcription factor HMGB1 to prevent inflammation. MiR-339-5p is involved in inflammatory regulation, and its regulatory mechanisms have not yet been elucidated.

In this study, we explored the effect of miR-339-5p on HMGB1. The dual-luciferase reporter assay indicated that miR-339-5p bound to the 3′-UTR of HMGB1. Western blotting and immunofluorescence staining confirmed that the miR-339-5p mimic decreased the expression of HMGB1 and that the miR-339-5p inhibitor increased the expression of HMGB1. These results suggest that the miR-339-5p/HMGB1 signalling pathway is important for the regulatory mechanisms involved in the inflammatory process and may be a key target in drug development for inflammatory disease treatment. To investigate the effects of paeonol and miR-339-5p on inflammation at different time points, we analysed the expression and secretion of inflammatory cytokines at 12 h and 24 h in LPS-stimulated RAW264.7 cells. Western blot, RT-qPCR and ELISA analyses indicated that miR-339-5p and paeonol inhibited the expression of the inflammation-related proteins HMGB1, TNF-α and IL-1β at the 12 h and 24 h time points in the LPS-induced RAW264.7 cells and that there was significant inhibition at the 24 h time point. Furthermore, compared with the LPS group, the miR-339-5p mimic transfection, siHMGB1-2 transfection, miR-339-5p mimic and siHMGB1-2 co-transfection, and miR-339-5p inhibitor and siHMGB1-2 co-transfection groups showed inhibited expression and secretion of HMGB1, TNF-α and IL-1β. However, there were no significant differences among the siHMGB1-2 transfection, miR-339-5p mimic and siHMGB1-2 co-transfection, and miR-339-5p inhibitor and siHMGB1-2 co-transfection groups. Our study confirmed that miR-339-5p inhibited inflammation and that this inhibition of the inflammatory response was dependent on HMGB1. We also used paeonol in combination with miR-339-5p. Western blot and ELISA analyses showed that compared with the LPS group, the miR-339-5p mimic transfection, paeonol treatment, miR-339-5p mimic transfection with paeonol treatment, and miR-339-5p inhibitor transfection with paeonol treatment groups exhibited downregulation production of TNF-α and IL-1β. The combination of paeonol and the miR-339-5p mimic inhibited TNF-α and IL-1β more significantly than the other treatments.

IKK-β is a serine-kinase that is stimulated by inflammatory cytokines. The phosphorylation of Ser177 and Ser181 in IKK-β leads to its activation; negative regulation of IKK-β can reduce the production of overactive inflammatory factors^54–56^. It has been reported that miR-339-5p downregulates inflammatory factor expression by inhibiting IKK-β activity^39^. Our study revealed that paeonol can inhibit IKK-β expression and increase miR-339-5p expression, suggesting that paeonol may upregulate miR-339-5p expression to inhibit the inflammatory response by targeting IKK-β. We further found that IKK-β expression was reduced after silencing HMGB1, while silencing IKK-β also reduced HMGB1 expression in LPS-stimulated RAW264.7 cells. These results indicated that there is positive feedback between HMGB1 and IKK-β in LPS-induced RAW264.7 cells.

The pathogenesis of sepsis is mainly excessive inflammation, resulting in multiple tissue and organ damage. When sepsis occurs, it will increase the release of pro-inflammatory cytokines and chemokines, causing and aggravating tissue damage, and even leading to multiple organ failur such as kidneys^57–59^. Inflammatory injury and ischemia are the main mechanisms of acute renal injury caused by sepsis. Studies have shown that in sepsis-induced acute kidney injury, a large number of inflammatory factors such as HMGB1 and TNF-α can be released in the kidney, resulting in renal tubular cell apoptosis and severe kidney injury^35^. Paeonol is a traditional Chinese medicine monomer anti-inflammatory agent^60,61^. It has been found that paeonol can inhibit the overproliferation of vascular smooth muscle cells (VSMCs) by inhibiting the expression of inflammatory factors TNF-α and IL-1β, and reduce local inflammation in AS^62^. Our previous studies have found that paeonol can inhibit the secretion or gene expression of inflammatory factors such as TNF-α and IL-1β and the transcriptional activity of NF-κB, and reduce the mortality of septic shock animals^33,42^. Therefore, this experiment used cecal ligation and puncture to establish CLP mice model, treated with paeonol, to explore the *in vivo* inflammatory effect of paeonol. The results showed that paeonol could significantly improve the survival rate of CLP mice, alleviate renal pathological damage, and inhibit the expression of inflammatory factors TNF-α and IL-1β.

In conclusion, our study showed that paeonol and miR-339-5p can inhibit inflammation. Paeonol inhibits the inflammatory response by upregulating miR-339-5p expression and subsequently downregulating HMGB1 and IKK-β expression. Furthermore, there is positive feedback between HMGB1 and IKK-β in LPS-induced RAW264.7 cells. Paeonol achieves multi-target and multi-pathway inhibition of the inflammatory response by upregulating miR-339-5p expression, which significantly enhances the inhibition of inflammation. *In vivo* experiments confirmed that paeonol could improve the survival rate of sepsis mice and protect the kidney of sepsis mice. This study also showed that paeonol and miR-339-5p may be promising therapeutic agents for the treatment of various inflammatory diseases such as Parkinson’s disease, organ failure, cancer, and especially sepsis, while HMGB1 and IKK-β may be promising therapeutic targets.

## Materials and Methods

### RAW264.7 cell culture

RAW264.7 cells, purchased from the Shanghai Institute of Cell Biology (Shanghai, China), were incubated in Dulbecco’s modified Eagle’s medium (DMEM) supplemented with 10% foetal bovine serum (FBS; Thermo Fisher Scientific, Inc.) and 1% antibiotics (100 U/mL penicillin and 100 mg/mL streptomycin; Thermo Fisher Scientific, Inc.) in a humidified, 5% CO_2_ and 37 °C environment. The medium was replaced every 3 days. For this study, cells were seeded in 6-well plates at 5 × 10^5^ per well, and the medium was changed to 10% FBS medium after 24 h. The control group was cultured with pure 10% FBS medium, the model group was subsequently stimulated with LPS (cat. no. L2880; Sigma-Aldrich; Merck KGaA, Darmstadt, Germany; 0.2 μg/mL) for 24 h, and the paeonol group was co-incubated with paeonol (1 mM; Shanghai YuanYe Biotechnology, Shanghai, China) and LPS (0.2 μg/mL) for 24 h.

### MiRNA microarray assay

Total RNA from RAW264.7 cells, including the cells in the LPS and paeonol groups, was extracted using TRIzol^®^ reagent (Thermo Fisher Scientific, Inc.) according to the manufacturer’s manual. A microarray assay was carried out by Guangzhou RiboBio Co., Ltd. (Guangzhou, China). For further analysis, all data were collected and sorted to identify the differentially expressed miRNAs according to fold change (|fc| ≥ 1.5). Multi Experiment Viewer 4.9.0 (MeV; Springer, Boston, MA, USA) was used to analyse the data.

### RT-qPCR

Total RNA was extracted as described above. Reverse transcription and qPCR were conducted following the manufacturer’s protocols for the Prime Script™ RT reagent kit and SYBR Premix EX Taq II kit (Takara Biotechnology Co., Ltd., Dalian, China). The ViiA 7 Real-time PCR System (Applied Biosystems; Thermo Fisher Scientific, Inc.) was used with the following reaction conditions: 95 °C for 10 s, 60 °C for 60 s and 95 °C for 15 s; 40 cycles. The 2^−ΔΔCq^ method was used to analyse the expression of miR-339-5p, and endogenous U6 expression was used for normalization. GAPDH was purchased from Songon Biotech (Shanghai, China) Co., Ltd. (cat. no. B661304; Shanghai, China). The primers were synthesized by Sangon Biotech Co., Ltd. (Shanghai, China) (Table 1).

**Table 1:** Primers for RT-qPCR.

Gene	Source	Sequence (5′-3′)
U6	Mouse	F: 5′-GCTTCGGCAGCACATATACTAAAAT-3′ R: 5′-CGCTTCACGAATTTGCGTGTCAT-3′
miR-339-5p	Mouse	GSP: 5′-GGGTCCCTGTCCTCCA-3′ R: 5′-CAGTGCGTGTCGTGGA-3′
IL-1β	Mouse	F: 5′-TCGCAGCAGCACATCAACAAGAG-3′ R: 5′-TGCTCATGTCCTCATCCTGGAAGG-3′
TNF-α	Mouse	F: 5’-ATGTCTCAGCCTCTTCTCATTC-3′ F: 5′-GCTTGTCACTCGAATTTTGAGA-3′

Note: GSP is a specific primer for the corresponding miRNA, and R is a primer that matches the RT primer.

### Dual-luciferase reporter assay

RAW264.7 cells were transfected with a luciferase reporter plasmid containing part of the HMGB1 3′-UTR. RAW264.7 cells at a density of 1 × 10^5^ per well were seeded in a 24-well plate until they reached 60% confluence. The pLUC-HMGB1-wild-type (WT) 3′-UTR or pLUC-HMGB1-mutant-type (MUT) 3′-UTR plasmid (Shenzen Huaan Pingkang Biological Technology Co., Ltd., Shenzhen, China) was co-transfected with the miR-339-5p mimic, miR-339-5p mimic negative control, miR-339-5p inhibitor or miR-339-5p inhibitor negative control (Guangzhou RiboBio Co., Ltd.) using Lipofectamine® 3000 (Thermo Fisher Scientific, Inc.). After 36 h of transfection, the firefly luciferase and Renilla luciferase activities were measured at 465 nm (Renilla luciferase) and 560 nm (firefly luciferase) according to the protocol of a dual-luciferase reporter assay kit (Promega Corporation, Madison, WI, USA), and relative fluorescence was determined by comparing with a negative control.

### Transient transfection of RAW264.7 cells with the miR-339-5p mimic, the miR-339-5p inhibitor, a negative control or an siRNA (siHMGB1 or siIKK-β)

Three HMGB1-specific siRNAs and three IKK-β specific siRNAs (siRNA-1, siRNA-2 and siRNA-3) were provided by Guangzhou RiboBio Co., Ltd. RAW264.7 cells were seeded in 6-well plates at 5 × 10^5^ cells per well until they reached 60% confluence. MiR-339-5p and a siRNA were co-transfected into RAW264.7 cells with Lipofectamine 3000 according to the manufacturer’s instructions. After 36 h, the silencing efficiencies of the siRNAs were analysed by Western blotting. The transfection concentration of the mimic, mimic negative control and siRNAs was 50 nM, and the transfection concentration of the inhibitor and inhibitor negative control was 100 nM. Western blot and immunofluorescence analyses were performed after 36 h.

### MiR-339-5p and siRNA transfections into LPS-stimulated RAW264.7 cells and experimental groupings

Following the transfection of miR-339-5p and a siRNA (siHMGB1 or siIKK-β) for 24 h, RAW264.7 cells were stimulated with LPS. RAW264.7 cells were pre-induced with 0.2 μg/mL LPS for 0.5 h before being treated with paeonol for 24 h. The experiment was designed with the following groups: (1) the normal control group without LPS stimulation, (2) the model group (LPS-induced cells), (3) the LPS + miR-339-5p mimic (with LPS treatment and transfection with the miR-339-5p mimic) group, (4) the LPS + miR-339-5p inhibitor (with LPS treatment and transfection with the miR-339-5p inhibitor) group, (5) the LPS + paeonol (with LPS and paeonol treatment) group, (6) the LPS + siRNA (with LPS treatment and transfection with an siRNA targeting HMGB1 or IKK-β) group, (7) the LPS + siRNA + miR-339-5p mimic (with LPS treatment and co-transfection with an siRNA targeting HMGB1 or IKK-β and the miR-339-5p mimic) group, and (8) the LPS + siRNA + miR-339-5p inhibitor (with LPS treatment and co-transfection with an siRNA targeting HMGB1 or IKK-β and the miR-339-5p inhibitor). RAW 264.7 cells were cultured in a 6-well plate at 5 × 10^5^ cells per well until they reached 60% confluence. MiR-339-5p and the appropriate siRNA were co-transfected into the RAW264.7 cells with Lipofectamine 3000 for 24 h. For the 0.2 μg/mL LPS-stimulated RAW264.7 cells, LPS was used to pre-induce RAW264.7 cells for 0.5 h before treatment with paeonol. Western blot and ELISA analyses were performed.

### Immunofluorescence assay

RAW264.7 cell samples were fixed with 4% paraformaldehyde at 37 °C for 30 min and permeabilized using 0.5% Triton X 100 for 15 min. Then, the RAW264.7 cells were blocked with 10% normal goat serum and incubated with a monoclonal rabbit anti-mouse HMGB1 antibody (1:400; cat. no. Ab184532; Abcam) at 4 °C overnight. This incubation was followed by an incubation with an Alexa Fluor 555-labelled anti-rabbit immunoglobulin G (IgG) secondary antibody (1:200; cat. no. A0453; Beyotime Institute of Biotechnology) for 1 h at 37 °C. An incubation with propidium iodide (PI) was conducted at 37 °C for 5 min, and the cells were examined by confocal microscopy (Olympus Corporation, Tokyo, Japan).

### Western blot analysis

Total protein was extracted using an extraction buffer (Thermo Fisher Scientific, Inc.). The proteins were separated by SDS-PAGE (12% acrylamide gel) and transferred onto a PVDF membrane. The membranes were subsequently blocked with 5% skim milk for 2 h at room temperature to inhibit nonspecific protein binding. This incubation was followed by an incubation with the primary antibodies anti-GAPDH (1:10,000; cat. no. AC002; ABclonal), anti-actin (1:1,000; cat. no. ab8226; Abcam), anti-HMGB1 (1:1,000; cat. no. Ab184532; Abcam), anti-TNF-α (1:1,000; cat. no. ab1793; Abcam), anti-IL-1β (1:1,000; cat. no. A1112; ABclonal), anti-pIKK-β (1:1,000; cat. no. Ab194519; Abcam) and anti-IKK-β (1:1,000; cat. no. A2087; ABclonal) at 4 °C overnight. The secondary antibodies were a horseradish peroxidase (HRP)-conjugated anti-rabbit IgG antibody (1:10,000; cat, no. ab6721; Abcam) and a horseradish peroxidase-conjugated anti-mouse IgG antibody (1:20,000; cat. no. AS003; ABclonal), which were incubated with the membranes for 1 h. The results were imaged and quantitated using Bio-Tanon Imagine (Tanon Science & Technology Co., Ltd., Shanghai, China). ImageJ software (version 1.4; National Institutes of Health, Bethesda, MD, USA) was used to analyse the specific protein band values of each group. All original pictures of western blot results are provided in supplementary file.

### Cytokine evaluation by ELISA

The concentrations of TNF-α and IL-1β in RAW264.7 cell supernatants were measured using ELISA kits (CUSABIO, Wuhan, China). Cell supernatants from different groups were analysed according to the ELISA kit protocols. The absorbance was determined at 450 nm using a Thermomax microplate reader (Bio-Tek, Winooski, VT, USA).

### Model of cecal ligation and puncture

Male C57BL/6 mice (6-8 weeks) were purchased from Guangdong Medical Laboratory Center (Guang dong, China). The animal experiment was carried out in accordance with the guidelines and regulations of China on use and care. The experimental animals are approved by the Animal Experimental Ethics Committee of Guangzhou University of Traditional Chinese Medicine. Anesthesia of mice by i.p. injecting 200 mg/kg ketamine and 10 mg/kg xylazine. Mice were subjected to sham or CLP surgery as previously described (Zhao *et al*., 2013). Paeonol was administered (120 mg/kg) i.p. to mice 30 min after CLP surgery. After 24 h, all mice were anesthetized and killed, kidneys were collected and fixed for hematoxylin and eosin (H&E) staining or stored at −80 °C for further western blot analysis. In addition, independent experiments were conducted to monitor the 9 days survival rate of mice in the same conditions.

### Statistical analysis

Data are presented as the mean ± standard deviation and were analysed using IBM SPSS 20 software (version 20; IBM corp., Armonk, NY, USA). Graphs were drawn using GraphPad Prism 7.0 software (version 7.0; GraphPad Software, Inc., La Jolla, CA, USA). Comparisons among groups were assessed using one-way analysis of variance (ANOVA). Comparisons between groups were made by analysing data with a post hoc test.

## Supplementary information

Supplementary Information
