# Supplementary material for: Paeonol attenuates inflammation by targeting HMGB1 through upregulating miR-339-5p
Source: Sci Rep. 2019 Dec 18;9:19370. doi: 10.1038/s41598-019-55980-4 (PMC6920373; doi:10.1038/s41598-019-55980-4)

Paeonol attenuates inflammation by targeting HMGB1 through upregulating miR-339-5p

Liyan Mei[a#], Meihong He[a#], Chaoying Zhang[a], Jifei Miao[a], Quan Wen[b], Xia Liu[c], Qin Xu[a], Sen Ye[a], Peng Ye[a], Huina Huang[a], Junli Lin[a], Xiaojing Zhou[a], Kai Zhao[a], Dongfeng Chen[a], Jianhong Zhou[a], Chun Li[d], Hui Li*[a]

Authors' information

[a] School of Basic Medical Sciences, Guangzhou University of Chinese Medicine, Guangzhou, Guangdong Province, 510006, China

[b] Guangdong-Hongkong-Macau Institute of CNS Regeneration, Jinan University, Guangzhou 510632, China

[c] School of Basic Medical Sciences, Guiyang University of Chinese Medicine, Guiyang, Guizhou Province, 550025, China

[d] School of Nursing Sciences, Guangzhou University of Chinese Medicine, Guangzhou, Guangdong Province, 510006, China

[#] Co-first authors contributed equally to this article.

Corresponding author

[*] Prof. Hui Li

Addresses: No. 232, East Waihuan Road, Guangzhou Higher Education Mega Center, Panyu District, Guangzhou, Guangdong Province, China.

E-mail: lihui@gzucm.edu.cn

Phone and fax numbers: (+86) 020-39358320

Figure 1

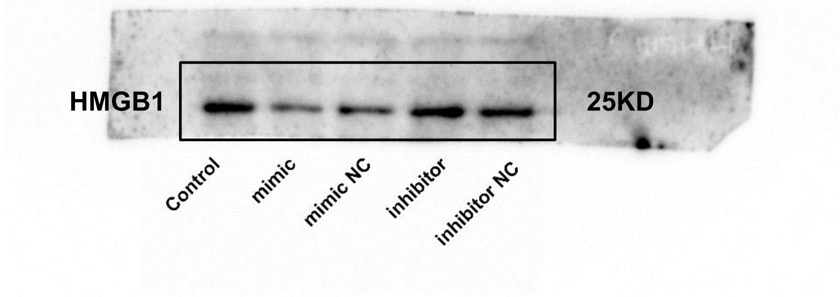

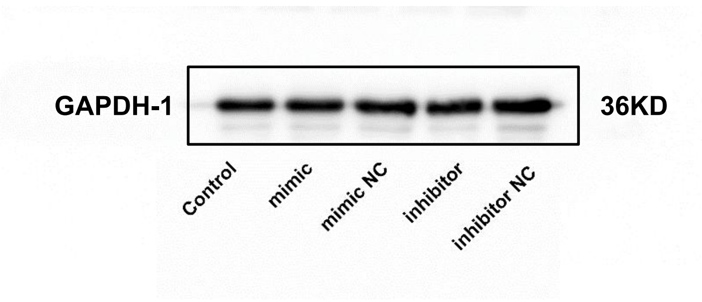

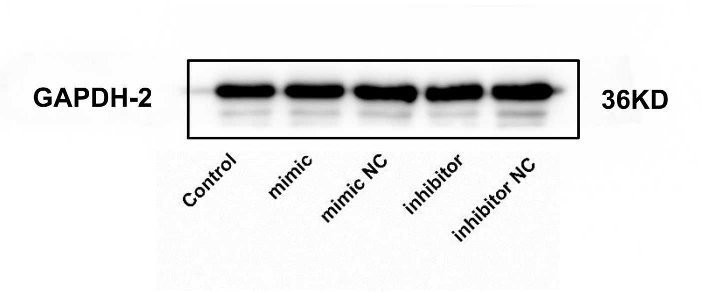

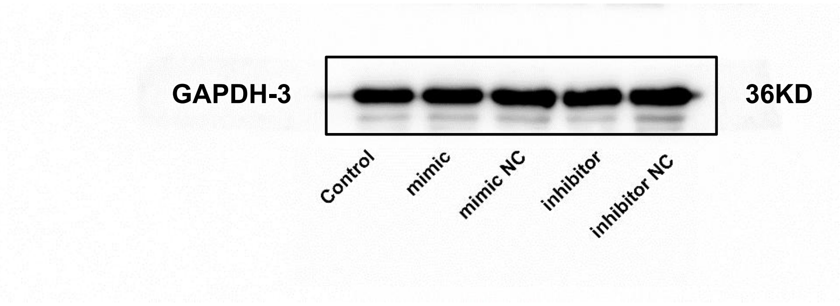

NOTE:
Full-length gels of HMGB1 and GAPDH are shown in Figure 1, the gel of control group was not put in the manuscript because we thought it is worthless under the condition that mimic NC and inhibitor NC existed.

GAPDH1-3 were the same gel with different exposure time.

All the gels/bots were derived from the same experiment.

Figure 2

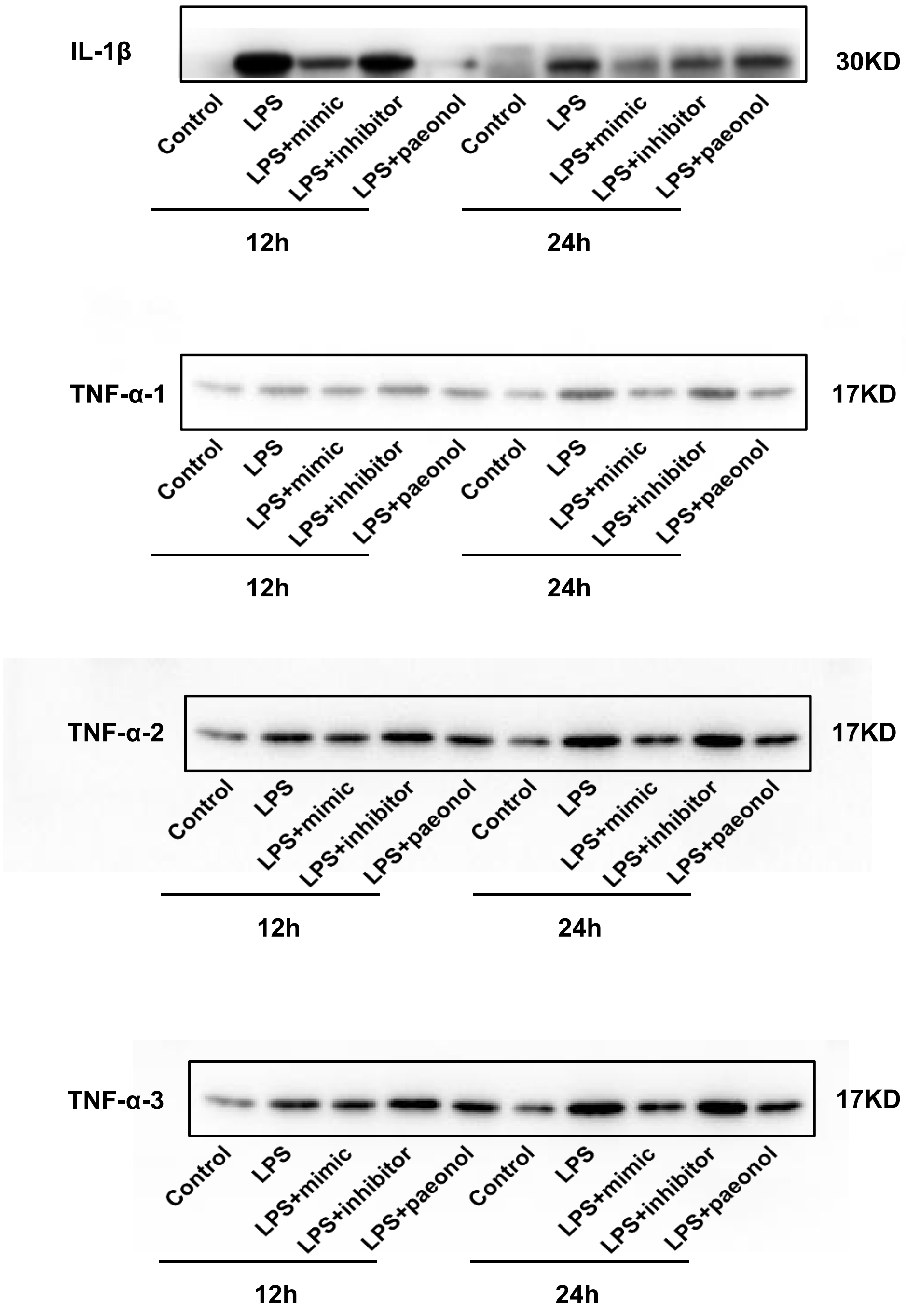

NOTE:
Full-length gels of HMGB1, TNF-α, IL-1β and β-actin are shown in Figure 2.

HMGB11-3, TNF-α1-3 and β-actin1-3 were the same gel with different exposure time.

All the gels/bots were derived from the same experiment.

Figure 2

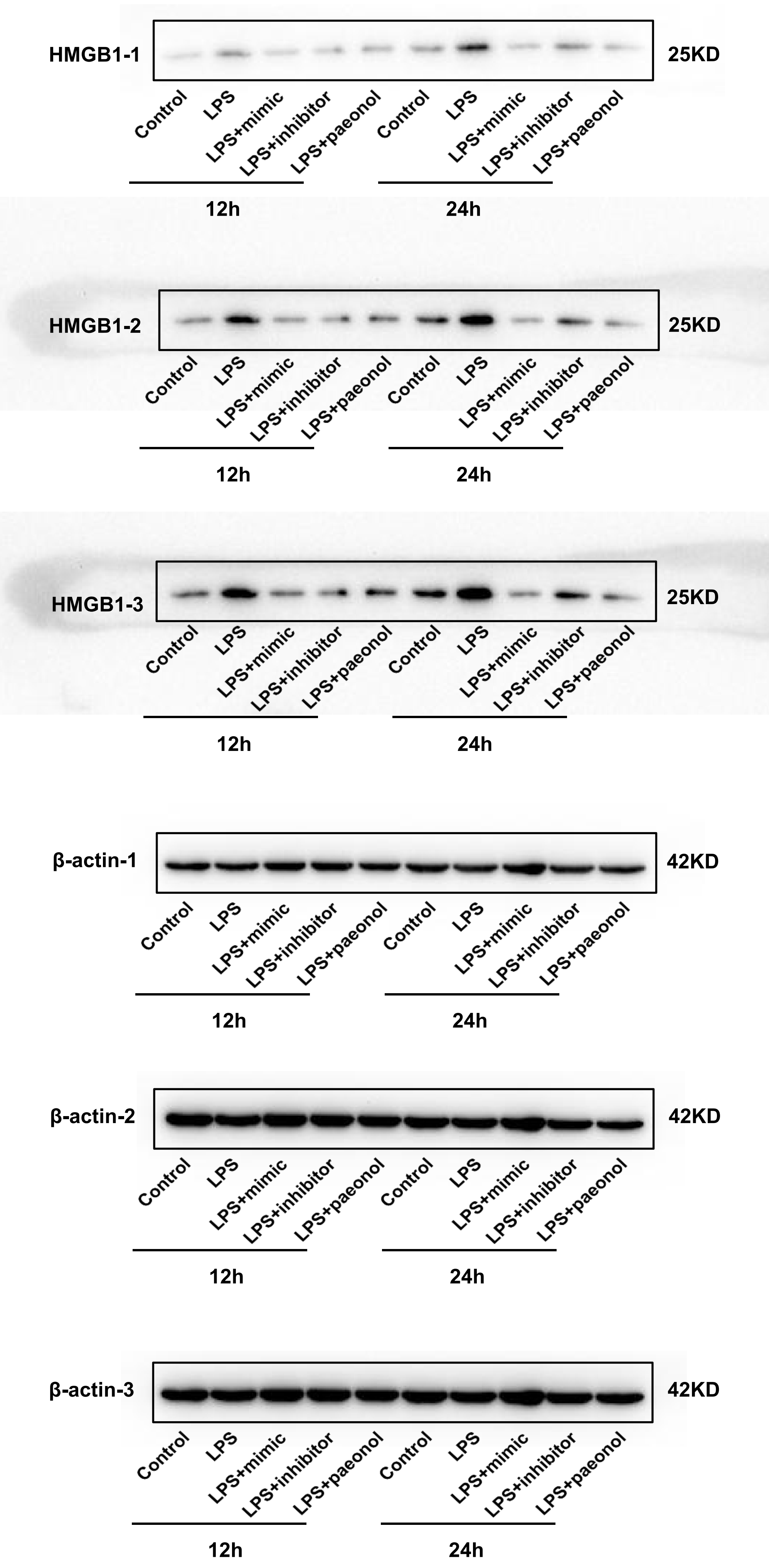

NOTE:
Full-length gels of HMGB1, TNF-α, IL-1β and β-actin are shown in Figure 2.

β-actin1-3 were the same gel with different exposure time.

All the gels/bots were derived from the same experiment.

Figure 3

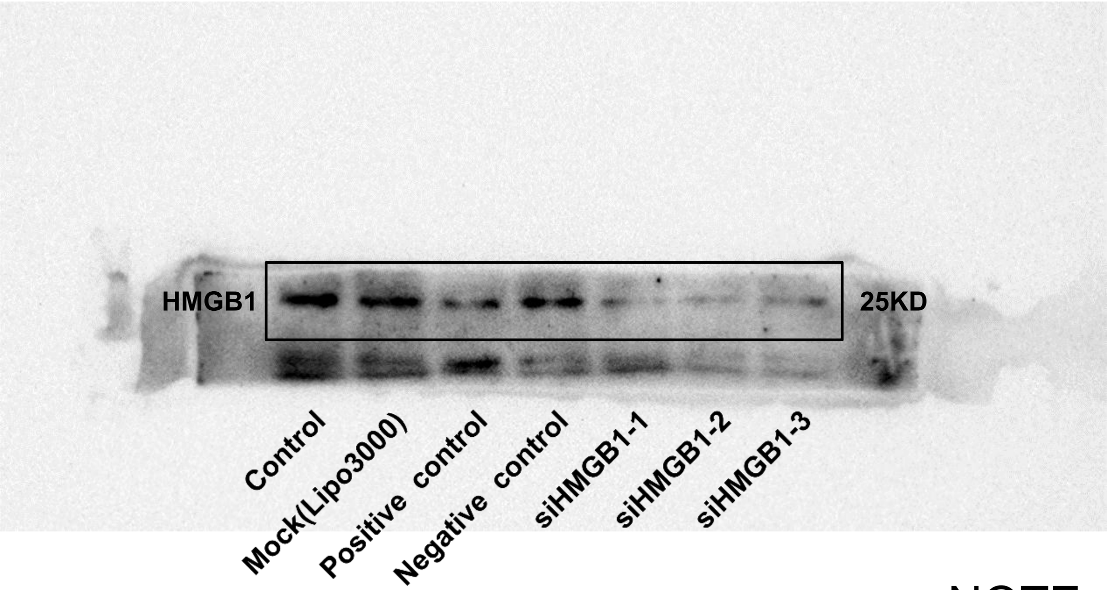

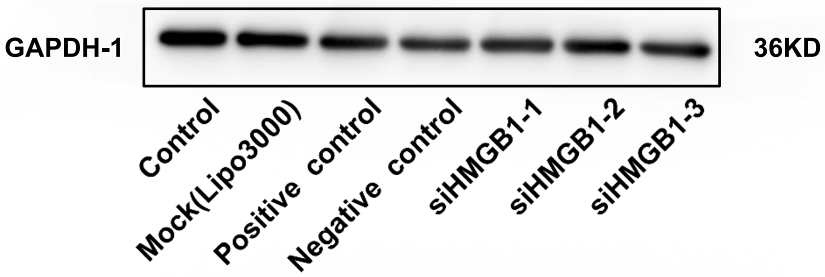

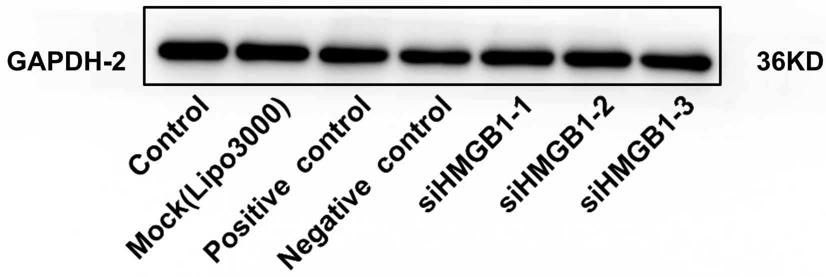

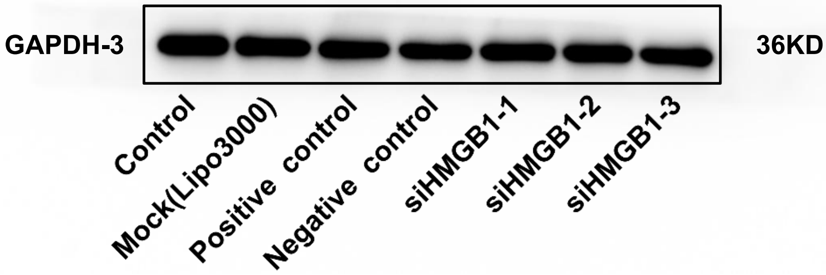

NOTE:
Full-length gels of HMGB1 and GAPDH are shown in Figure 3, the gels we cropped is HMGB1 and the gels below maybe other proteins belong to HMGB family.

GAPDH1-3 were the same gel with different exposure time.

All the gels/bots were derived from the same experiment.

Figure 4

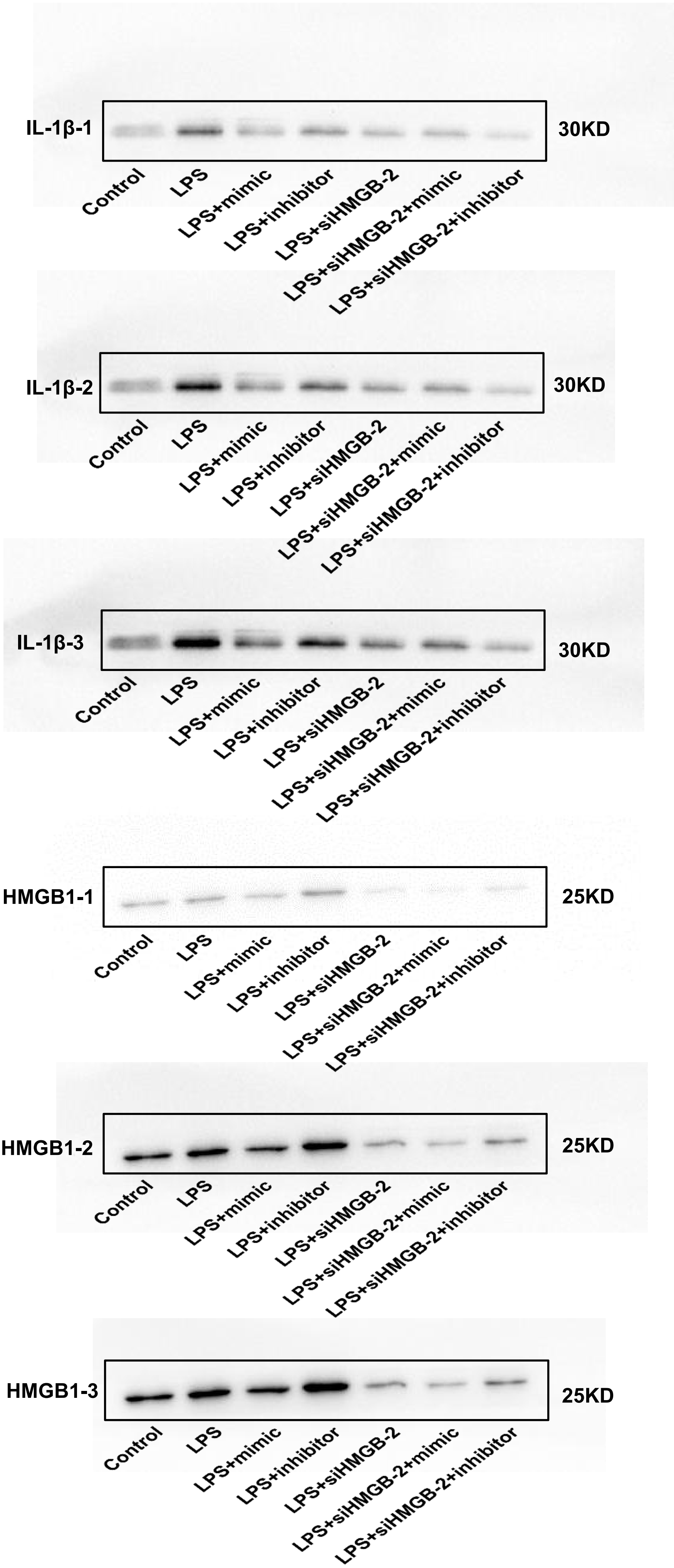

NOTE:
Full-length gels of HMGB1, TNF-α, IL-1β and GAPDH are shown in Figure 4.

HMGB11-3, TNF-α1-3, IL-1β1-3 and GAPDH1-3 were the same gel with different exposure time.

All the gels/bots were derived from the same experiment.

Figure 4

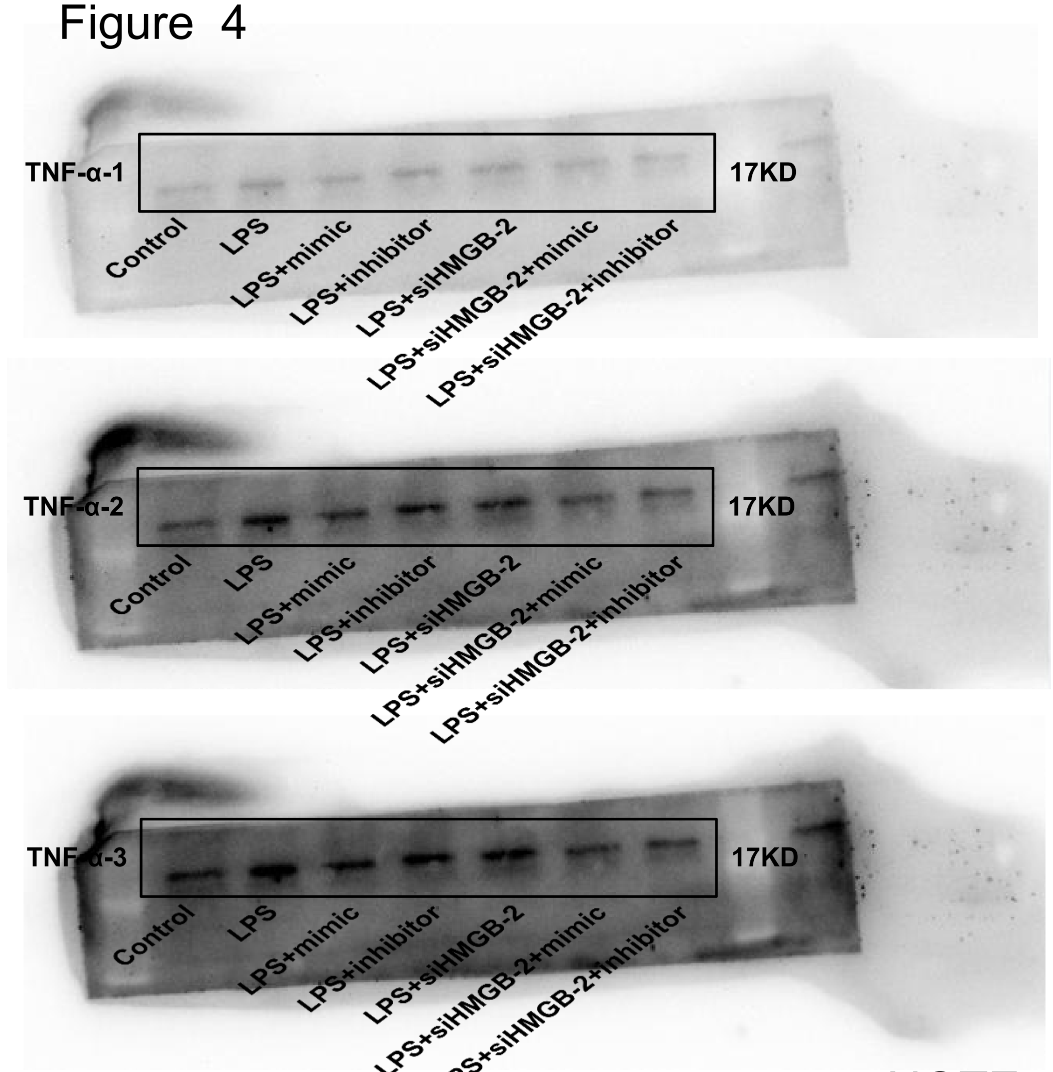

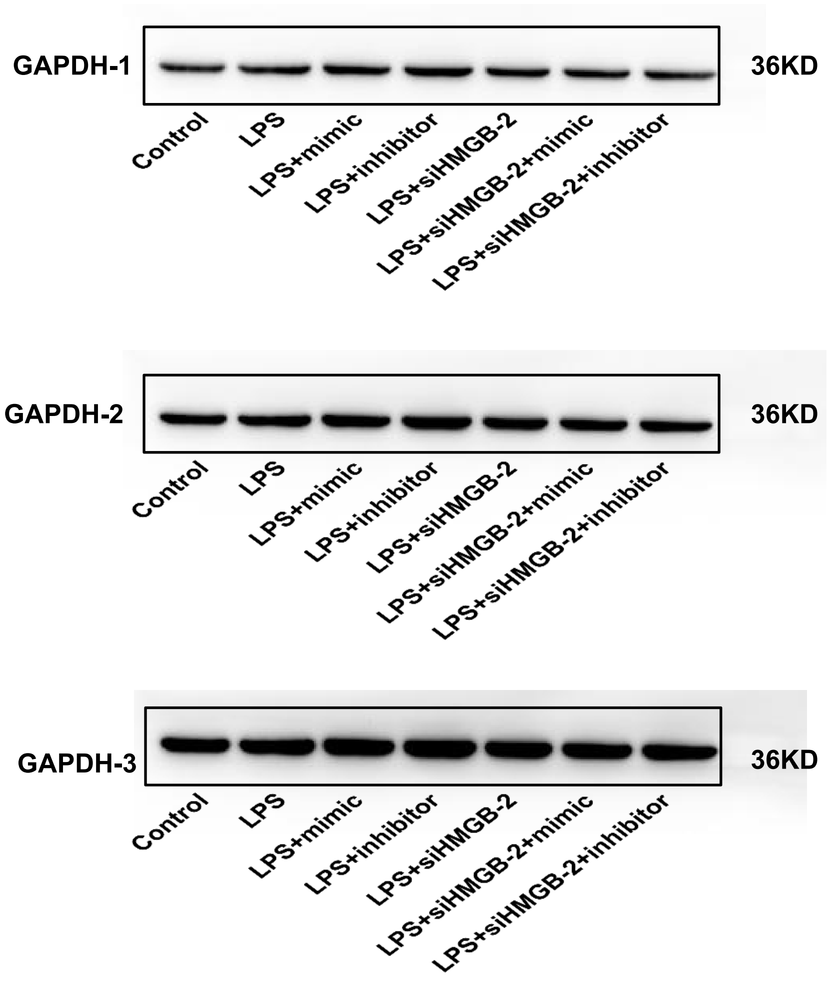

NOTE:
Full-length gels of HMGB1, TNF-α, IL-1β and GAPDH are shown in Figure 4.

HMGB11-3, TNF-α1-3, IL-1β1-3 and GAPDH1-3 were the same gel with different exposure time.

All the gels/bots were derived from the same experiment.

Figure 5

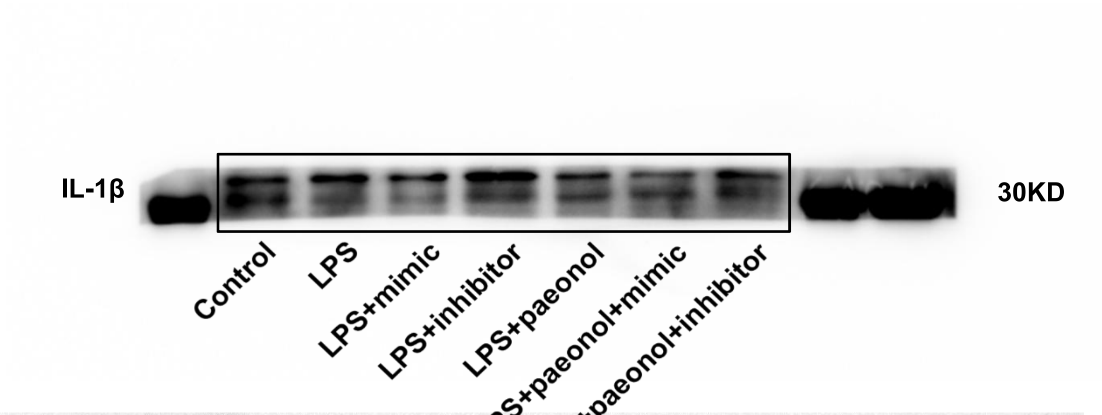

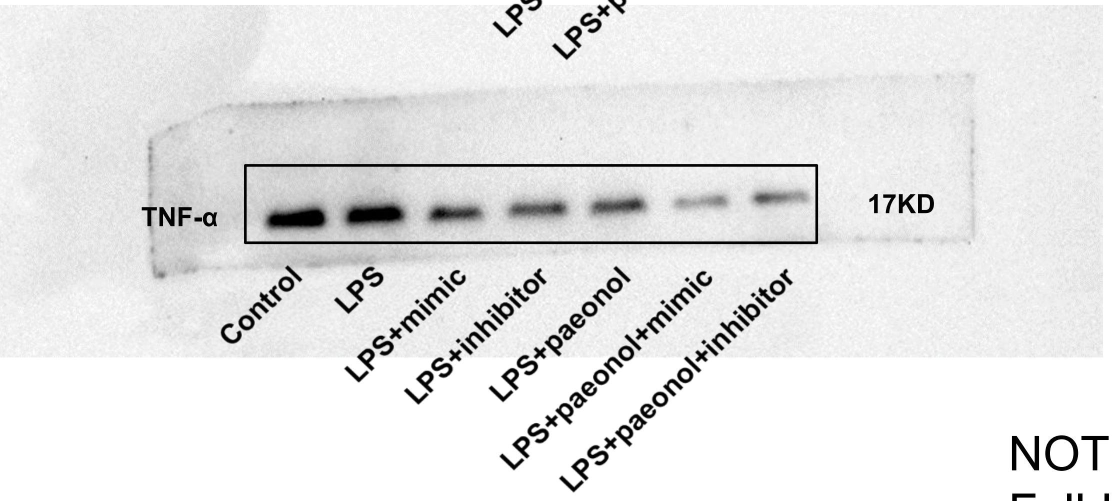

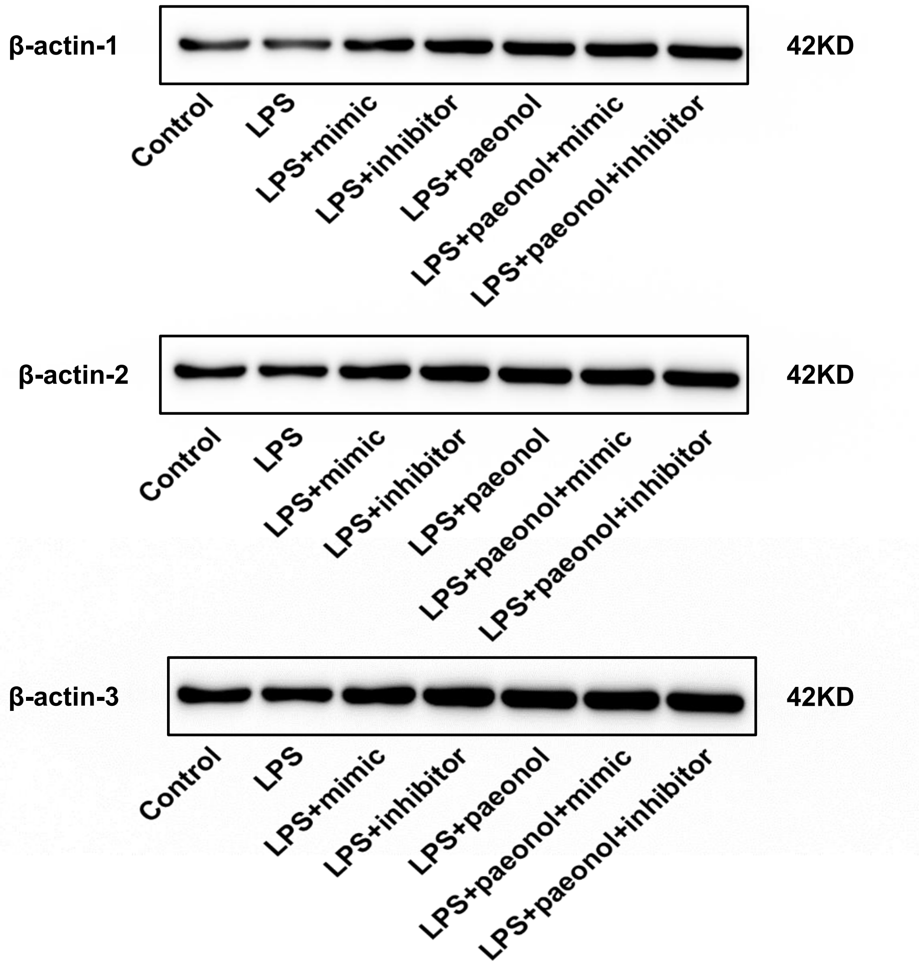

NOTE:
Full-length gels of TNF-α, IL-1β and β-actin are shown in Figure 5.

β-actin1-3 were the same gel with different exposure time.

All the gels/bots were derived from the same experiment.

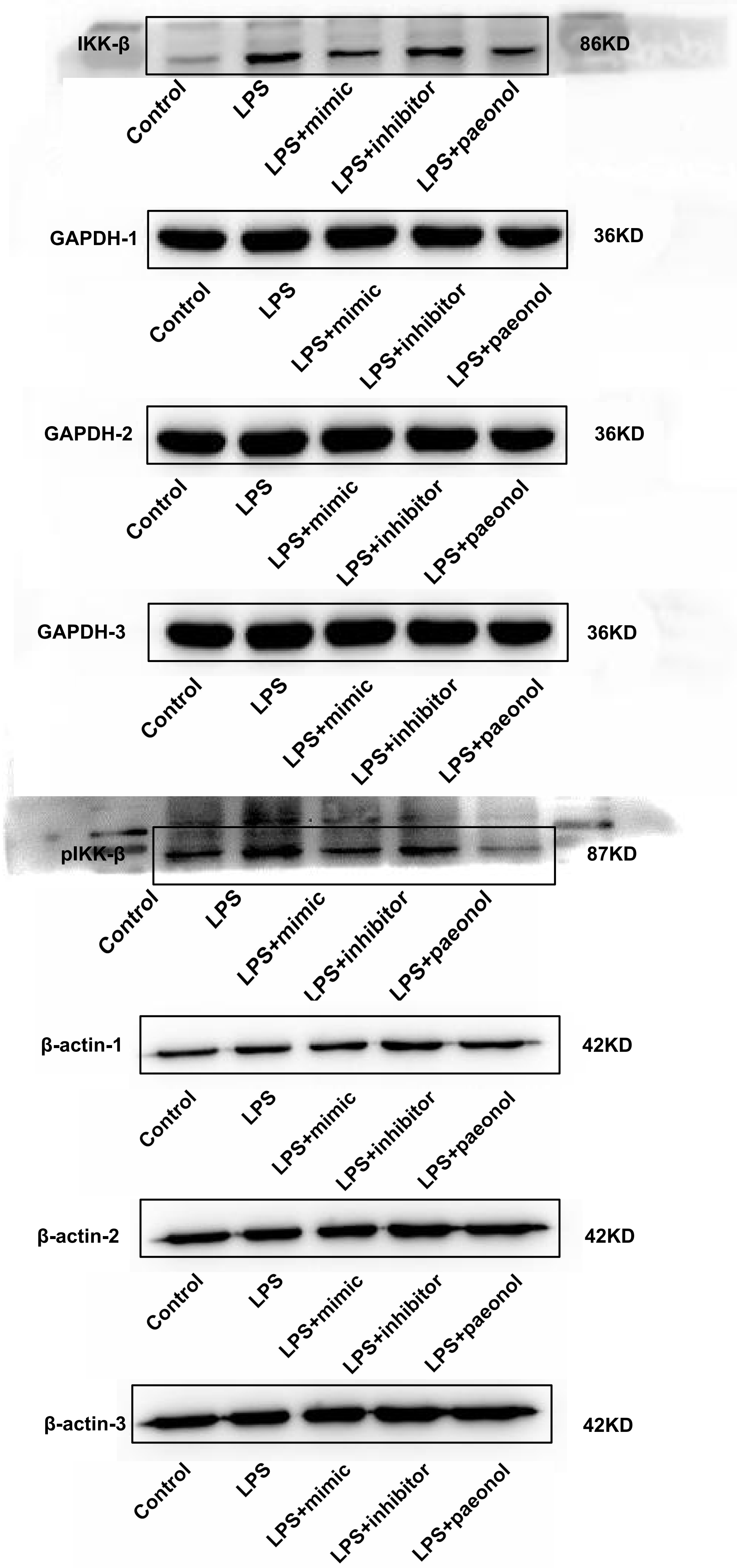

NOTE:
Full-length gels of IKK-β GAPDH, pIKK-β and β-actin are shown in Figure 6.

GAPDH1-3 and β-actin1-3 were the same gel with different exposure time.

All the gels/bots were derived from the same experiment.

Figure 7

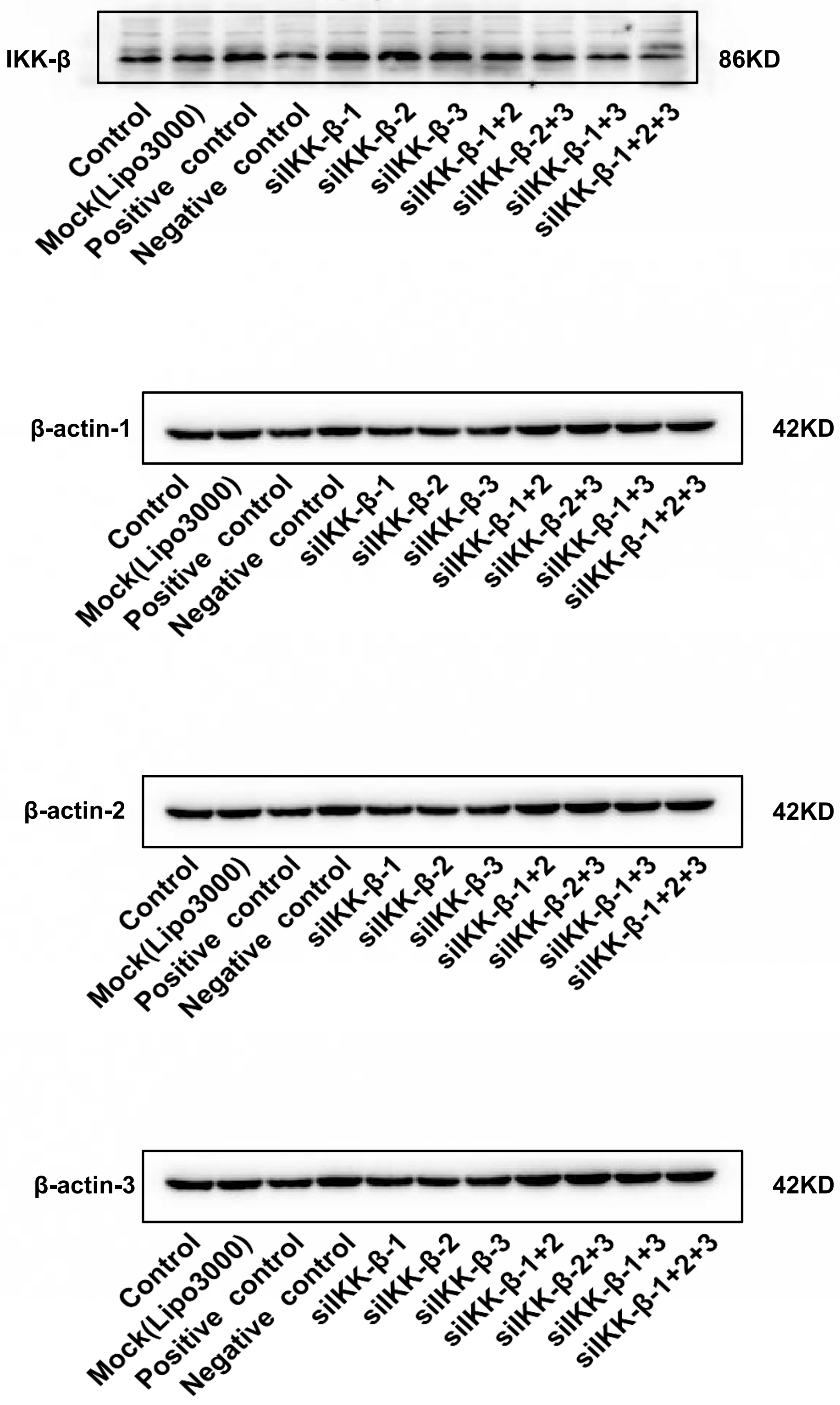

NOTE:
Full-length gels of IKK-β and β-actin are shown in Figure 7.

β-actin1-3 were the same gel with different exposure time.

All the gels/bots were derived from the same experiment.

Figure 8

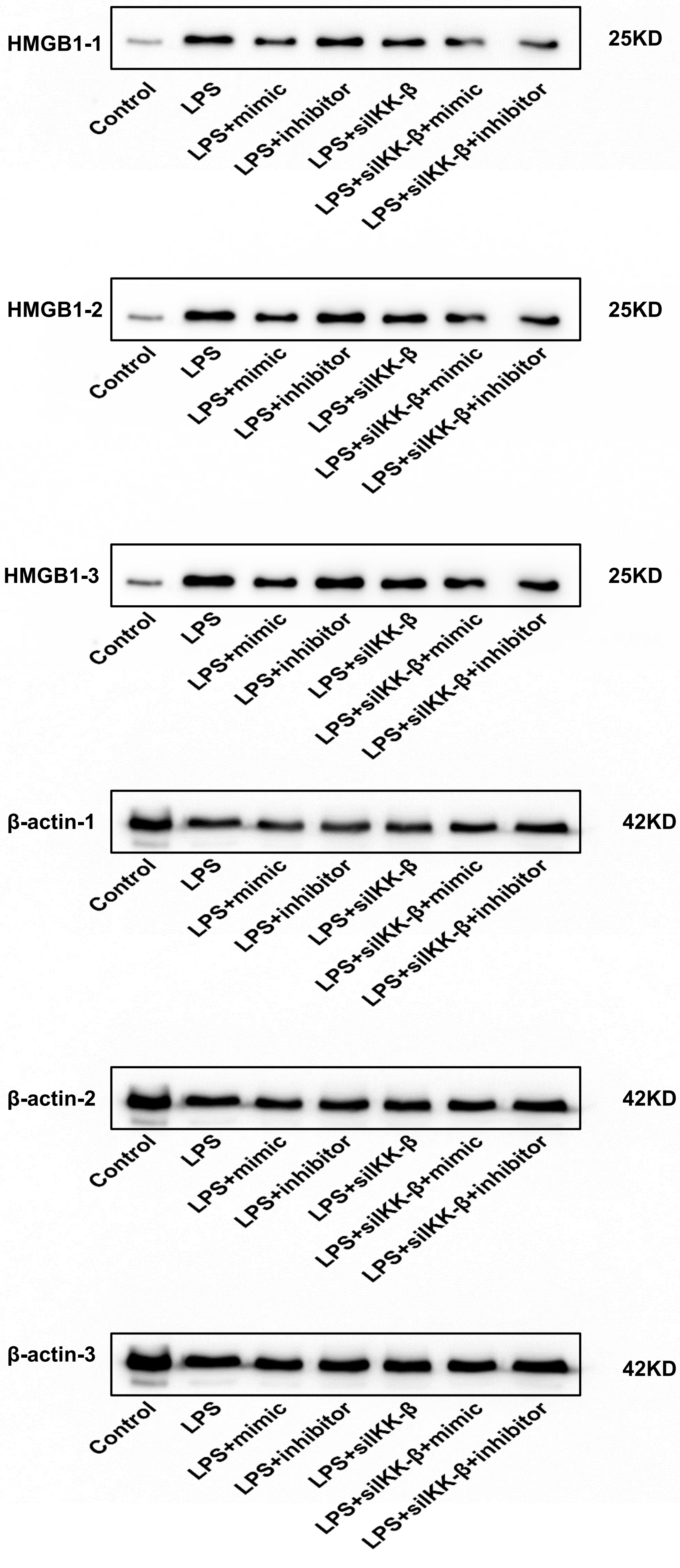

NOTE:
Full-length gels of HMGB1 and β-actin are shown in Figure 8.

HMGB11-3 and β-actin1-3 were the same gel with different exposure time.

All the gels/bots were derived from the same experiment.

Figure 9

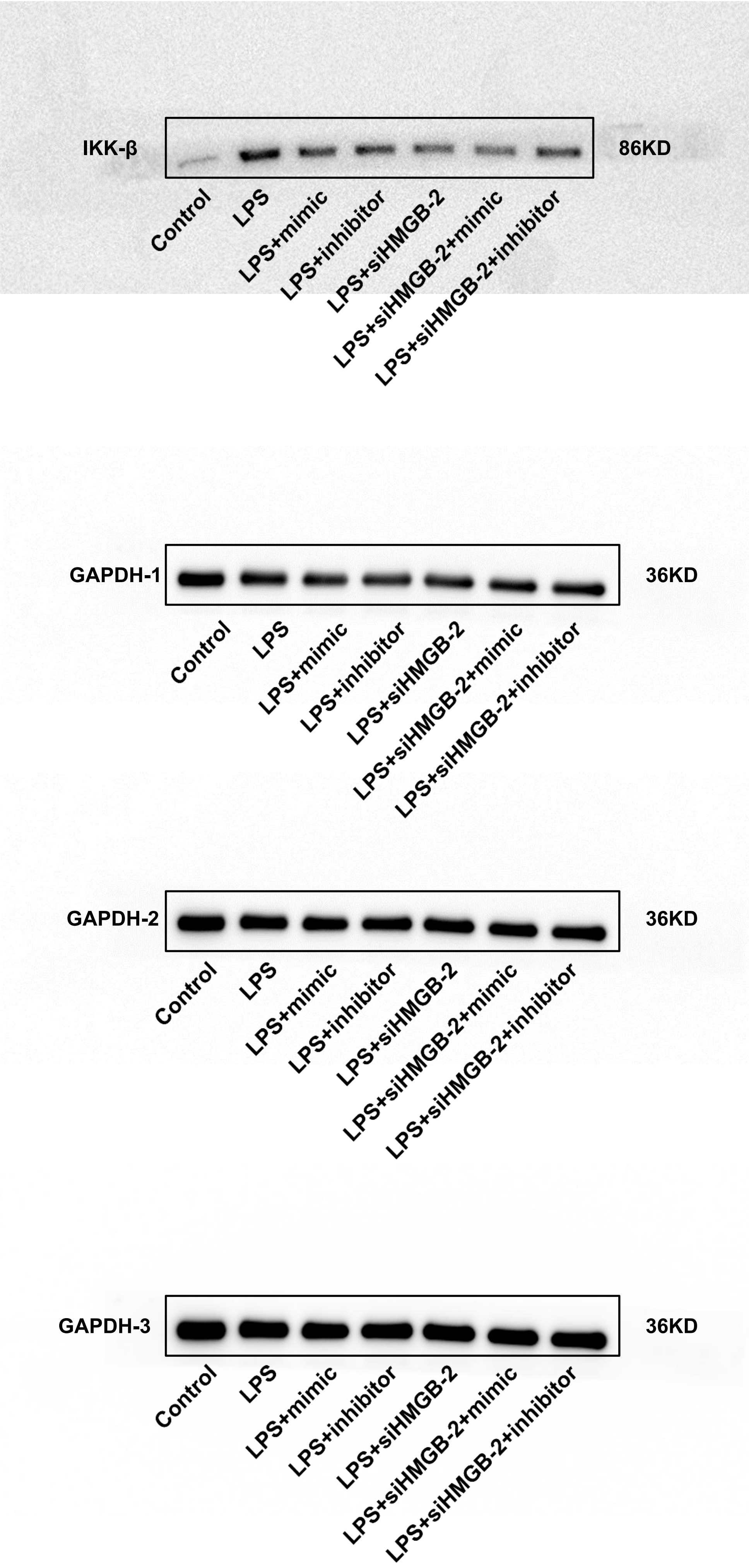

NOTE:
Full-length gels of IKK-β and GAPDH are shown in Figure 9.

GAPDH1-3 were the same gel with different exposure time.

All the gels/bots were derived from the same experiment.

Figure 10

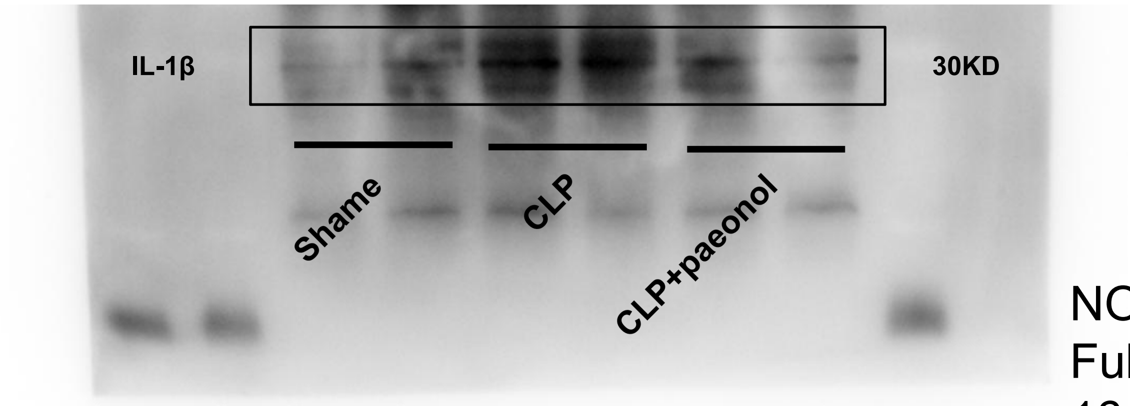

NOTE:
Full-length gels of IL-1β, TNF-α and GAPDH are shown in Figure10.

All the gels/bots were derived from the same experiment.

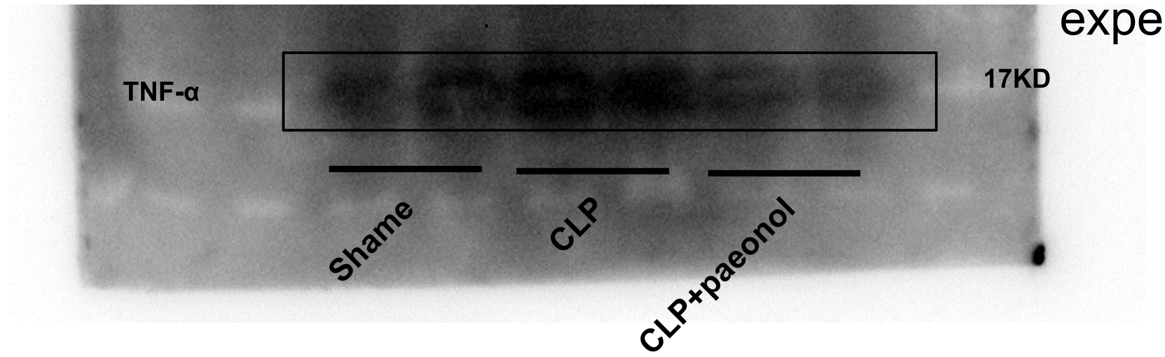

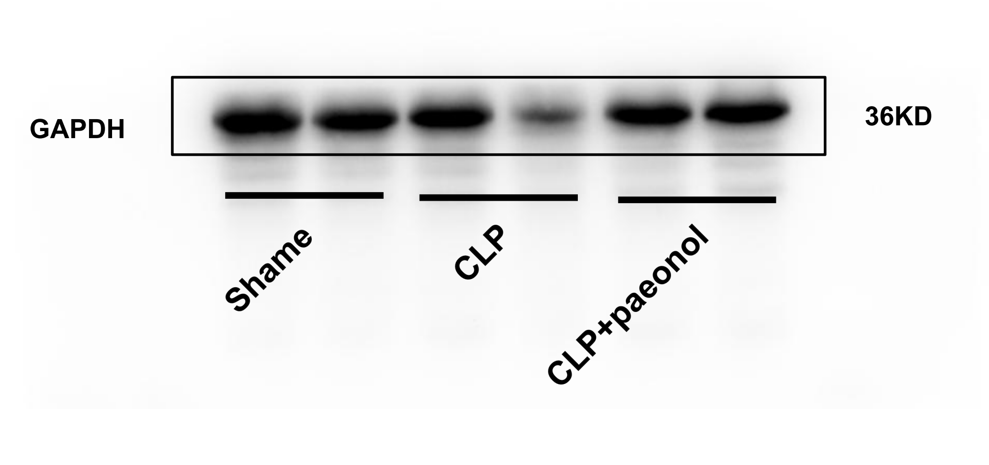

Supplement: Supplementary file 1 — Supplementary Information [file 41598_2019_55980_MOESM1_ESM.pdf]
